# Novel Enyne-Modified 1,4-Thiazepines as Epidermal Growth Factor Receptor Inhibitors: Anticancer and Computational Studies

**DOI:** 10.1021/acsomega.4c07877

**Published:** 2024-12-26

**Authors:** Harika Atmaca, Çisil Çamlı Pulat, Suleyman Ilhan, Elif Serel Yilmaz, Metin Zora

**Affiliations:** † Department of Biology, Faculty of Engineering and Natural Sciences, Manisa Celal Bayar University, 45140 Manisa, Turkey; ‡ Applied Science Research Center, Manisa Celal Bayar University, 45140 Manisa, Turkey; § Department of Chemistry, Middle East Technical University, 06800 Ankara, Turkey

## Abstract

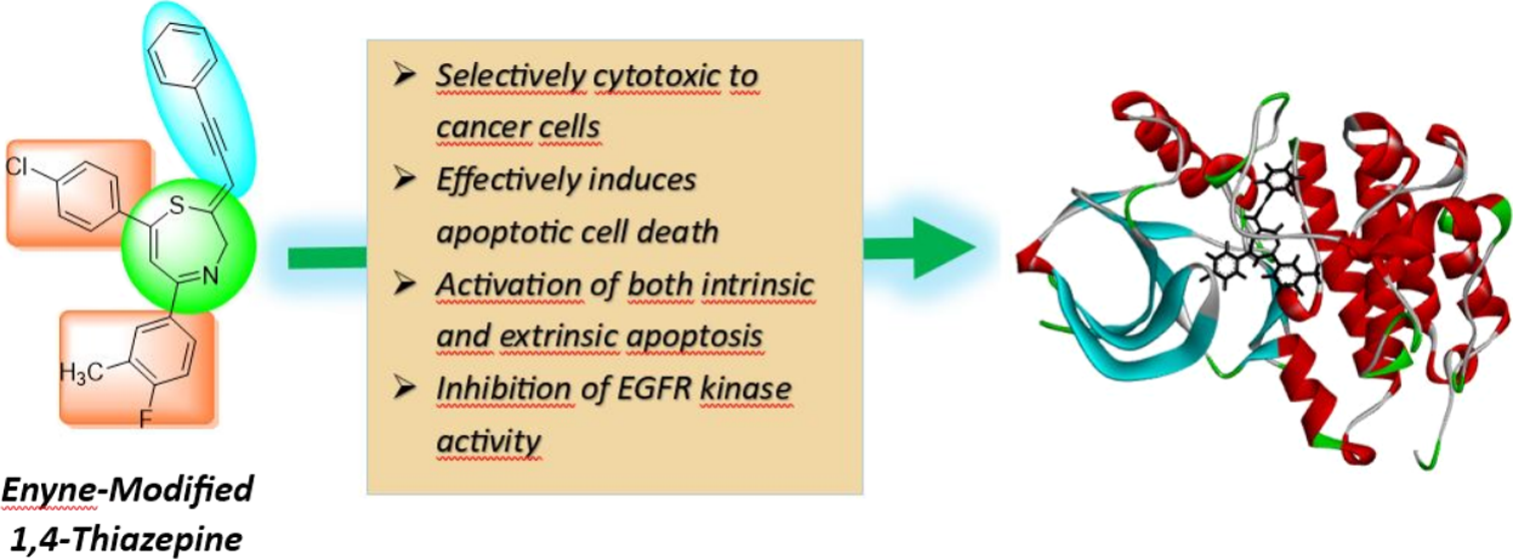

1,4-Thiazepines (TZEPs) featuring enyne modifications represent promising candidates in cancer therapy. We synthesized novel TZEP derivatives and assessed their cytotoxicity, apoptosis induction, EGFR inhibition, and molecular interactions. TZEPs exhibited cytotoxic effects against cancer cell lines, with compounds TZEP6 and TZEP7 showing significant activity. Flow cytometry analysis revealed TZEP7-induced apoptosis across various cancer types. RT-qPCR analysis demonstrated downregulation of antiapoptotic Bcl-2, upregulation of pro-apoptotic Bax, and increased caspase levels following TZEP7 treatment. Additionally, TZEP7 inhibited EGFR kinase activity in cancer cells, with molecular docking confirming strong binding affinities to EGFRWT and mutant EGFRT790M. AdmetSAR analysis indicated favorable pharmacokinetic properties for TZEP7. These findings underscore the potential of enyne-modified TZEPs as selective cytotoxic agents with apoptotic and EGFR inhibitory activities, highlighting their significance in cancer therapy.

## Introduction

Cancer is a significant global health issue due to its high prevalence, increasing incidence, and substantial mortality rates. It affects millions of people worldwide and stands as the second leading cause of death.^1^ Cancer is a complex disease group that arises from abnormal cell growth and proliferation. Various types of cancer exist, each originating from different tissues or organs in the body. While there are distinct differences among other types of cancer, they share fundamental characteristics related to abnormal cell growth, genetic alterations, invasion, metastasis, and evading apoptotic cell death.^2^

Genetic alterations, whether inherited or acquired mutations, are pivotal in initiating and driving cancer development. These mutations can disrupt normal cellular processes, including DNA repair mechanisms and cell cycle control, allowing cancer cells to evade growth inhibition and apoptosis.^3^ EGFR, which stands for Epidermal Growth Factor Receptor, is a cell surface receptor belonging to the ErbB family of receptor tyrosine kinases. Aberrant activation of EGFR signaling is commonly observed in various cancers due to mutations in the EGFR gene or gene amplification. These alterations can lead to constitutive activation of EGFR signaling, promoting uncontrolled cell growth and tumor progression and inhibiting apoptotic processes.^4^ Activated EGFR signaling can suppress apoptosis by increasing the expression of antiapoptotic proteins, such as Bcl-2 and Bcl-xL, and decreasing the expression or activity of pro-apoptotic proteins like Bax and Bak. Mutations within the EGFR kinase ATP-binding domain, such as in-frame deletions of exon 19 and the L858R mutation, are known oncogenic drivers. Additionally, the T790 M mutation, often termed the secondary “guardian” mutation, enhances ATP binding affinity and is common across different cancer types.^5^ As a result, EGFR has emerged as a crucial therapeutic target in cancer treatment.

Traditional cancer treatments like chemotherapy and radiation therapy often come with severe side effects. These treatments do not discriminate between cancerous and healthy cells, leading to widespread damage. This highlights the urgent need for novel chemotherapeutic drugs that are more effective and cause fewer side effects.^6^ Researchers focus on developing targeted therapies that specifically attack cancer cells while sparing normal cells, thereby reducing side effects and improving treatment efficacy.

**Figure 1:**
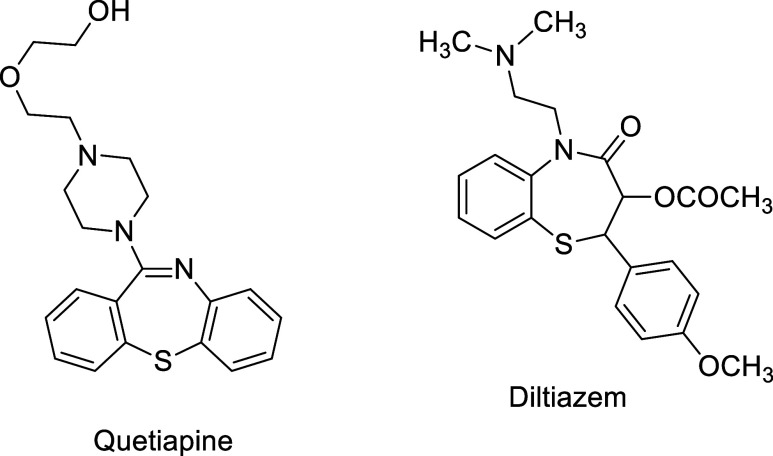
Examples of 1,4-thiazepine-bearing drugs.

1,4-Thiazepines (TZEPs) are a class of heterocyclic compounds containing a seven-membered ring with one sulfur atom and one nitrogen atom.^7,8^ Their unique chemical structure and the diverse biological activities they exhibit make TZEPs attractive targets for drug discovery and development. For instance, antipsychotic *Quetiapine* is used in the treatment of schizophrenia, acute mania and depression related bipolar disorders,^9^ and cardiovascular and antiarrhythmic *Diltiazem* is effective in blocking calcium channels,^10^ the structures of which are shown in Figure 1. Continued research into the synthesis, structure–activity relationships, and pharmacological properties of 1,4-thiazepines holds promise for identifying novel therapeutic agents across various medical conditions.^11^ Due to their unique chemical structure and versatile biological activities, researchers have extensively explored the medicinal chemistry of TZEPs to develop new drugs with improved therapeutic profiles. Structural modifications of the core scaffold have led to the synthesis of analogs with enhanced potency, selectivity, and pharmacokinetic properties. Some derivatives have been investigated as potential lead compounds for treating various diseases, including cancer, infectious diseases, and neurological disorders.

The enyne moiety, combined with alkene and alkyne functionalities, is a crucial structural element in organic synthesis and drug design. Its unique reactivity and presence in biologically active compounds make it an important subject of study in chemistry and pharmacology.^12^ We hypothesized that incorporating the essential structural features of 1,4-thiazepines with a conjugated enyne moiety could generate compounds with pronounced or distinct biological properties while also allowing for their conversion into more complex structures. Recently, we have synthesized 12 new 1,4-thiazepine derivatives containing enyne moiety, namely 2-(prop-2-yn-1-ylidene)-2,3-dihydro-1,4-thiazepines (TZEPs), as shown in Table 1.^13^ The synthesis of TZEPs were accomplished by the reaction of *N*-(2,4-pentadiynyl) β-enaminones (NPE) with Lawesson’s reagent in refluxing benzene (For details, see the Supporting Information).^13^ Since TZEPs represent a promising class of compounds in the search for effective cancer treatments, we now aim to evaluate the apoptotic and antiproliferative activities of these synthesized 1,4-thiazepine derivatives bearing enyne moiety (Table 1). Notably, β-enaminones are also valuable building blocks and pharmacophores in drug development.^14^ The potential of TZEPs to inhibit EGFR, a pivotal player in many cancers, makes them valuable candidates for drug development. By disrupting EGFR signaling pathways, 1,4-thiazepine derivatives can reduce tumor growth and promote apoptosis, offering hope for improved treatment options with fewer side effects and enhanced efficacy against resistant cancer strains.

**Table 1:**
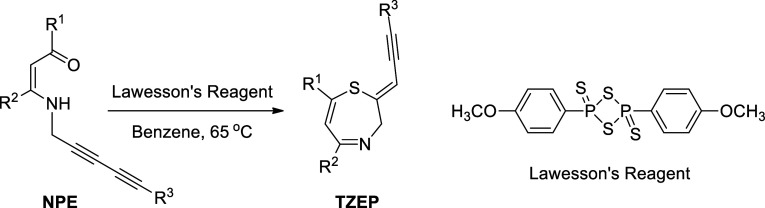

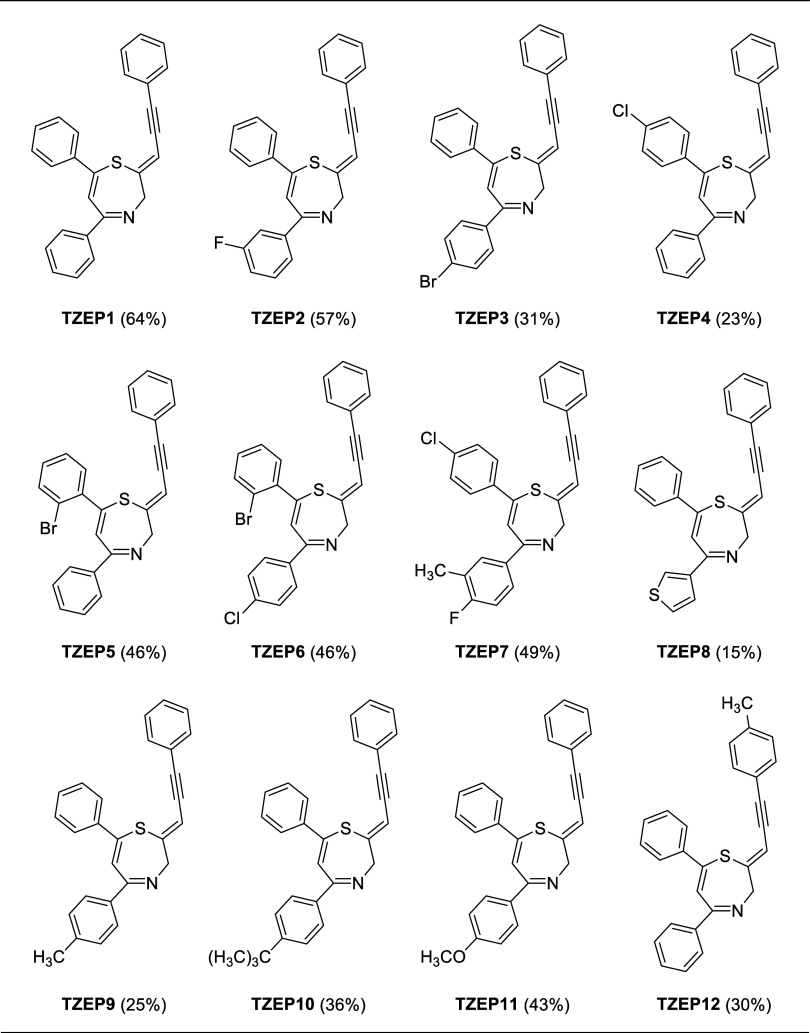
Scope of the Synthesis of 1,4-Thiazepines (TZEPs)

## Materials and Methods

### Cell Culture and Viability

Human cell lines for breast cancer (MCF-7), lung cancer (A549), prostate cancer (PC-3), and nontumorigenic HEK-293 were sourced from the Health Protection Agency (U.K.) and the Interlab Cell Line Collection (Italy). The cells were cultured in RPMI medium (Sigma) supplemented with 10% heat-inactivated fetal bovine serum, 1% penicillin, and 1% l-glutamine, and incubated in a humidified CO_2_ atmosphere at 37 °C.

To evaluate the cytotoxic effects of TZEPs, which were dissolved in dimethyl sulfoxide (DMSO), the XTT assay (2,3-bis(2-methoxy-4-nitro-5-sulfophenyl)-2*H*-tetrazolium-5-carboxanilide) was employed. Cells were plated in 96-well plates at a density of 10^4^ cells per well and exposed to varying concentrations of TZEPs (ranging from 1 to 250 μM) for 24, 48, and 72 h. Following the incubation, 100 μL of XTT was added to each well, and the plates were incubated for an additional 4 h at 37 °C. The resulting color change was measured by recording the absorbance at 570 nm using a microplate reader (Tecan).

### Calculation of Selectivity Index (SI)

The IC_50_ values, which indicate the concentration of the compound required to inhibit 50% of cancer cell proliferation, were determined using Biosoft CalcuSyn 2.1 software. To assess the selectivity of TZEPs toward cancer cells compared to normal cells (HEK-293), the selectivity index (SI) was calculated using the formula
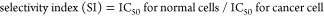
An SI greater than 1 indicates that the drug is more toxic to target cells than normal cells, while an SI of 1 suggests equal toxicity to both target and normal cells. Conversely, an SI less than 1 means the drug is more toxic to normal cells than to target cells, which is undesirable in therapeutic contexts. In the literature, a selectivity index of 2 or higher is generally considered significant, implying that the compound selectively targets diseased cells with relatively low toxicity to healthy cells.^15^

### Apoptosis Detection Using Flow Cytometry

Annexin V, a protein with a high affinity for phosphatidylserine (PS), is pivotal in detecting apoptosis. Normally, PS is located in the inner leaflet of the plasma membrane, but during apoptosis, it moves to the outer leaflet, making it a key marker for apoptotic cells.^16^ Annexin V binds specifically to PS on the surface of these cells. A secondary dye like propidium iodide (PI) is used to distinguish apoptotic cells from viable ones with intact membranes. We can identify and quantify viable cells (annexin V–, PI–) and apoptotic cells (annexin V+, PI+) by analyzing fluorescence patterns. Here, we employed the FITC Annexin V Apoptosis Detection Kit I from BD Pharmingen. Cells were seeded at a density of 10^6^ cells per well in a 6-well plate and exposed to IC_50_ concentration of TZEP7 for 72 h. Following treatment, cells were rinsed with cold PBS, resuspended in 1 mL of 1× Binding Buffer, and stained with 5 μL of Annexin V FITC and 5 μL of PI. After vortexing, the samples were incubated at room temperature (25 °C) for 15 min in the dark. Postincubation, 400 μL of 1× Binding Buffer was added to each sample, and apoptosis was measured using a BD Accuri C6 Flow Cytometer. Cisplatin was used as an apoptosis-inducing reference drug.

### Quantitative Real-Time PCR (qRT-PCR) Analysis

To measure the mRNA expression levels of apoptosis-related genes B-cell lymphoma 2 (Bcl-2), Bcl-2-associated X protein (Bax), Cyclin-dependent kinase 2 (CDK2), and Cyclin E, total mRNA was extracted from cells treated with compound 6f using Trizol reagent (Sigma). cDNA was synthesized from the total mRNA using the Qiagen cDNA synthesis kit according to the manufacturer’s instructions. The following primers, each at a concentration of 350 nM, were employed: Bcl-2; forward (GGTGCCACCTGTGGTCCACCTG), reverse (CTTCACTTGTGGCCCAGATAG); Bax; forward (ATGGACGGGTCCGGGGAGCAGC), reverse (CCCCAGTTGAAGTTGCCGTCAG); Caspase-8; forward (CAAACTTCACAGCATTAGGGAC), reverse (ATGTTACTGTGGTCCATGAGTT); Caspase-9; forward (TGTCTACGGCACAGATGGA), reverse (GGACTCGTCTTCAGGGGA); EGFRWT: forward (TGAGGTGACCCTTGTCTCTG), reverse (CCTGTGCCAGGGACCTTAC−); EGFRT790 M forward (CGAAGGGCATGAGCTGCATGATGAGCTGCACGGTGG), reverse (CCACCGTGCAGCTCATCATGCAGCTCATGCCCTTC); human GAPDH; forward (GGCAAATTCAACGGCACAGT), reverse (AGATGGTGATGGGCTTCCC). The PCR mix was prepared with 1× buffer containing 0.2 mM dNTPs, 1.6 mM MgCl2, 50 mmol of each primer set, cDNA, and 0.25 U Taq polymerase. The PCR conditions were as follows: an initial denaturation at 95 °C for 60 s, followed by 50 cycles of 95 °C for 15 s, 60 °C for 15 s, and 72 °C for 60 s. The cycle threshold (CT) for each replicate was normalized to the average CT of the endogenous control (GAPDH). The relative quantification of gene expression was calculated using the comparative CT method.^17^

### Inhibition Assay of EGFR Kinase Activity

The inhibitory effects of the target compounds on various EGFR kinases were assessed using a homogeneous time-resolved fluorescence (HTRF) assay, employing the HTRF KinEASE-TK kit (Cat# 62TK0PEC, Cisbio).^18^ TZEP7 was tested at a range of diluted concentrations in the presence of 1% dimethyl sulfoxide (DMSO), with the kinase and compounds preincubated for 5 min. The reactions were initiated by adding ATP and TK-substrate-biotin, followed by a 60 min incubation at room temperature. The reactions were then stopped using a stop buffer containing 62.5 nM Strep-XL665 and TK antibody (Ab)-Cryptate. After a 1 h incubation of the plate, readings were taken on a microplate reader under standard HTRF conditions. IC_50_ values were calculated using GraphPad Prism 5.0 software. Each reaction was duplicated, and at least three independent determinations were conducted. The data were analyzed using GraphPad Prism.

### Molecular Docking Studies of TZEP7

Molecular docking studies for TZEP7 were performed targeting the ATP binding sites of both wild-type EGFR tyrosine kinase (EGFRWT) and the mutant form (EGFRT790M) using Autodock Vina 4.2.5.1 software. The X-ray crystallographic structures of the EGFR proteins (EGFRWT PDB ID: 4HJO and EGFRT790 M PDB ID: 3W2O) were sourced from the RCSB Protein Data Bank (https://www.rcsb.org/) in PDB format.

The Protein Preparation Wizard was utilized to ready the proteins by assigning bond orders, adding hydrogen atoms, processing metals, and removing water molecules. Energy minimization was performed with a root-mean-square deviation (RMSD) of 0.30 Å. The three-dimensional (3D) structures of the ligands were created using Maestro 8.5, part of the Schrödinger suite, while Open Babel software was used to generate the 3D structures of the synthesized compounds. The grid box parameters were optimized to achieve the lowest RMSD value below 2 Å, with the grid center for EGFRWT set at *X* = 21.41, *Y* = 3.62, and *Z* = 21.94, and the grid box dimensions at 60 Å × 60 Å × 60 Å. These parameters were consistently applied for EGFRT790 M after calibration and optimization. The setup generated various docked conformations, which were then visualized using Discovery Studio software to examine the secondary structures of the molecules. Erlotinib (EB) was used as an EGFR inhibitor reference drug.

### AdmetSAR Analysis

The AdmetSAR 2.0 online tool (http://lmmd.ecust.edu.cn/admetsar2) was employed to forecast the absorption, distribution, metabolism, excretion, and toxicity characteristics of TZEP7. This platform provides a range of physicochemical properties, including molecular weight (M.W.), log *P*_o/w_ c (Octanol–water partition coefficient), solubility (log *S*), skin permeation (log *K*_p_), hydrogen bond acceptor (Hy-A), hydrogen bond donor (Hy-D), total polar surface area, and molar refractivity (Lipinski). These parameters furnish valuable insights into the ADMET properties of any drug or organic compound. Compliance with Lipinski’s rule of five (Ro5) and other criteria is indispensable when developing a molecule as a potential drug candidate. According to Ro5, a compound’s ADME parameters signify its accessibility within the body. Notably, meeting the following criteria is essential: molecular weight ≤500, hydrogen bond acceptor ≤10, hydrogen bond donor ≤5, log *P* ≤ 5, molar refractivity ≤140, adherence to the rule of five, and falling within the log *P*_o/w_ range of −2 to 6.5, polar surface area range of 7 to 200, log *S* range above −4, and a drug score value above 0.5 are deemed acceptable for the synthesized compounds.^19^

### Statistical Analysis

Statistical analysis was conducted using GraphPad Prism 5.0 software. Initially, a one-way ANOVA test was employed, followed by Tukey’s post-ANOVA test for multiple comparisons, with a significance level set at *p* < 0.05. The data are expressed as mean ± standard deviation (SD).

## Results and Discussion

### In Vitro Evaluation of the Cytotoxic Activity of the Synthesized TZEPs

1,4-Thiazepines are currently the focus of attention of medicinal chemists due to their structural flexibility, advanced biological activities, and the possibility of creating derivatives with specific interactions with molecular targets such as enzymes, receptors, and ion channels.^7,8,13^ Moreover, thiazepine and thiazepinone derivatives are of pharmacological importance with potential applications in cancer treatment, especially due to the important role of the C–S bond present in thiazepines in their anticancer activity.^20^ Anticancer activities of these compounds have been demonstrated in many studies.^20,21^ The enyne moiety, a structural unit in organic compounds featuring a triple bond adjacent to a double bond within a carbon chain, has garnered significant interest in medicinal chemistry due to its versatile reactivity and potential pharmacological properties. In the context of anticancer compounds, the incorporation of the enyne moiety into molecular structures has shown promise in the development of novel agents with cytotoxic activity against cancer cells. Researchers have synthesized various enyne-containing compounds and evaluated their anticancer potential through in vitro and in vivo studies. These compounds often exhibit selective cytotoxicity toward cancer cells while sparing normal cells, making them attractive candidates for further development as anticancer therapeutics.^22^

In our previous study, we synthesized novel 1,4-thiazepine compounds with a conjugated enyne moiety that are likely to have cytotoxic and apoptotic activities.^13^ Using the conventional XTT method, all the synthesized TZEPs were tested for their potential cytotoxic activity against a panel of human cancer cell lines with overexpressed EGFR. The data in Table 2 showed that TZEP derivatives exhibited cytotoxic effects on the tested cell lines. Although the compounds were generally less effective than cisplatin against the tested cancer cell lines, TZEP6 (IC_50_ values of 15.5 ± 2.8, 14.2 ± 2.2, and 12.4 ± 3.6 μM) and TZEP7 (IC_50_ values of 14.1 ± 2.1, 12.7 ± 2.0, and 16.9 ± 1.6 μM) demonstrated significant activity against MCF-7, A549, and PC-3 cancer cells, respectively, compared to cisplatin (IC_50_ values of 14.7 ± 0.6, 13.4 ± 1.4, and 12.8 ± 2.8 μM). According to Table 2, TZEP1 was effective against PC-3 prostate cancer cells, and TZEP3 showed a similar effect to cisplatin against MCF-7 cancer cells; however, they were not evaluated as effective in other cancer types and therefore were not selected for further detailed experiments.

**Table 2:** IC_50_ Values (μM) of 1,4-Thiazepines (TZEP1-12) and Reference Drugs on a Panel of Human Cancer Cells and Non-Tumorigenic Cells (*TZEP with Selectivity Index of >2)

TZEPs	non-tumorigenic cells (HEK-293)	breast cancer (MCF-7)	lung cancer (A549)	prostate cancer (PC-3)
TZEP1	29.5 ± 0.8	69.4 ± 1.2	38.7 ± 3.2	12.4 ± 3.2
TZEP2	45.7 ± 2.7	66.4 ± 1.8	61.4 ± 1.6	57.7 ± 1.4
TZEP3	35.5 ± 1.3	13.3 ± 1.0	60.3 ± 0.8	71.2 ± 0.9
TZEP4	47.3 ± 0.7	81.2 ± 4.4	71.1 ± 2.6	73.5 ± 2.2
TZEP5	20.4 ± 1.4	22.3 ± 1.8	45.8 ± 0.9	54.9 ± 3.2
TZEP6	10.9 ± 3.4	15.5 ± 2.8	14.2 ± 2.2	12.4 ± 3.6
TZEP7*	54.3 ± 0.9	14.1 ± 2.1	12.7 ± 2.0	16.9 ± 1.6
TZEP8	99.2 ± 3.0	85.7 ± 2.4	112.8 ± 0.8	95.4 ± 1.2
TZEP9	98.8 ± 0.7	78.4 ± 1.8	96.6 ± 1.4	102.7 ± 2.0
TZEP10	89.0 ± 0.5	76.3 ± 0.8	64.4 ± 1.8	73.7 ± 3.2
TZEP11	112.4 ± 3.0	98.2 ± 2.8	107.9 ± 2.6	81.6 ± 3.4
TZEP12	110.3 ± 1.2	92.6 ± 1.0	90.5 ± 0.7	100.7 ± 3.0
erlotinib	12.9 ± 3.0	13.4 ± 2.0	18.4 ± 0.4	15.5 ± 30.8
cisplatin	15.0 ± 0.7	14.7 ± 0.6	13.4 ± 1.4	12.8 ± 2.8

Derivatives containing the enyne moiety, particularly those incorporating Cl, displayed heightened cytotoxicity in our study. The increased effectiveness may result from the unique structural characteristics of the enyne moiety within the 1,4-thiazepine framework, which could facilitate interactions with crucial biological targets implicated in cancer progression. Second, the conjugated system of the enyne moiety might enhance the compound’s reactivity, leading to improved cancer cell targeting and cytotoxicity.^23^ Additionally, derivatives with Cl may engage in specific molecular interactions within cancer cells, such as with enzymes, receptors, or signaling pathways, thereby augmenting their cytotoxic and apoptotic activities. Moreover, the chemical structure of Cl-containing derivatives might influence their bioavailability and metabolic stability, ensuring better penetration into cancer cells and prolonged retention, ultimately enhancing their efficacy.^24^

Fluorine substitution has been extensively studied in drug research as a method to enhance biological activity and improve chemical or metabolic stability. The presence of a fluorine atom often enhances the ability to cross cell membranes, thereby increasing bioavailability. Additionally, due to its small size and high electronegativity, the fluorine atom can affect the electron distribution in the attached molecules, resulting in stronger and more specific interactions with target proteins.^25^ Finally, synergistic effects between Cl, F, the 1,4-thiazepine scaffold, and the enyne moiety may further amplify the compound’s anticancer properties. Further experimental investigations could provide deeper insights into the specific mechanisms underlying the observed enhancements in cytotoxicity and apoptotic activity.

In drug design, the Selectivity Index (SI) is a crucial parameter that measures the therapeutic window of a compound. It helps to determine how selectively a drug targets pathogenic cells, such as cancer cells, over normal, healthy cells. A high SI indicates a compound effective against target cells with minimal toxicity to normal cells, making it a desirable characteristic for a therapeutic agent.^15^ We also calculated the SI for TZEP6 and TZEP7, effective against all the tested cancer cell lines (Table 3). SI values of TZEP6 were 0.70, 0.76, and 0.87 for MCF-7, A549, and PC-3 cells, respectively, while they were 3.85, 4.27, and 3.21 for TZEP7. TZEP7, which had a high SI index value and was found to be selectively cytotoxic to cancer cells, was chosen for further experiments.

**Table 3:** SI Values of 1,4-Thiazepines (TZEP1-12) and Reference Drugs^a^

TZEPs	breast cancer (MCF-7)	lung cancer (A549)	prostate cancer (PC-3)
TZEP1	0.42	0.76	2.37
TZEP2	0.68	0.74	0.79
TZEP3	2.66	0.58	0.49
TZEP4	0.58	0.66	0.64
TZEP5	0.91	0.44	0.37
TZEP6	0.70	0.76	0.87
TZEP7	3.85	4.27	3.21
TZEP8	1.15	0.87	1.03
TZEP9	1.26	1.02	0.96
TZEP10	1.16	1.38	1.20
TZEP11	1.14	1.04	1.37
TZEP12	1.19	1.21	1.09
erlotinib	0.96	0.70	0.83
cisplatin	1.02	1.11	1.17

a An SI greater than 1 means the drug is more toxic to target cells than to normal cells, while an SI of 1 indicates equal toxicity for both. An SI less than 1 suggests the drug is more harmful to normal cells, which is not ideal. A selectivity index of 2 or higher is typically considered significant, indicating that the compound effectively targets diseased cells while minimizing harm to healthy cells.

**Figure 2:**
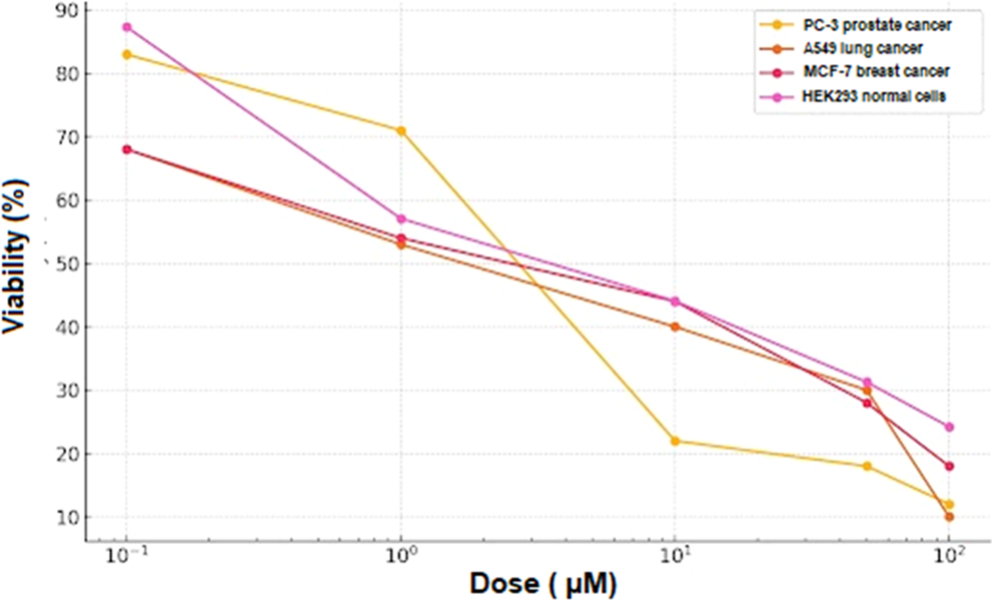
Dose–response curve for the different cell lines (PC-3 prostate cancer, A549 lung cancer, MCF7 breast cancer, and HEK293 normal cells). The *x*-axis represents the dose on a logarithmic scale, and the *y*-axis represents cell viability as a percentage. Each curve shows how the viability of the different cell lines decreases as the dose increases.

As shown in Figure 2, the dose–response curve for PC-3 cells shows a sharp decline in viability as the dose of TZEP7 increases. At the highest concentration (100 μM), viability was reduced to 12%, indicating a strong cytotoxic effect. At 50 μM, cell viability remained low at 18%, and a substantial reduction in viability was still observed at 10 μM (22%). The lower doses of 1 and 0.1 μM saw viability at 71 and 83%, respectively, which indicates that concentrations below 10 μM might not be sufficient to induce strong cytotoxic effects.The A549 lung cancer cell line shows even greater sensitivity to TZEP7 compared to PC-3 cells, with an IC_50_ of 12.09 μM. At 100 μM, the viability of A549 cells plummeted to 10%, and even at 50 μM, viability dropped to 30%. At 10 μM, viability stood at 40%, indicating significant cytotoxicity, with the effect tapering off at 1 μM (53%) and 0.1 μM (68%).The MCF-7 breast cancer cells exhibit a similar dose–response pattern to A549 cells, though with a slightly higher IC_50_ value of 14.21 μM. At 100 μM, MCF-7 cell viability dropped to 18%, and at 50 μM, viability remained at 28%. However, the viability at 10 μM was higher than in A549 cells, at 44%, suggesting a somewhat reduced sensitivity to TZEP7. At 1 and 0.1 μM, viability was 54 and 68%, respectively. HEK293 cells, representing normal healthy cells, show significantly higher viability at all concentrations tested compared to cancer cell lines. At 100 μM, cell viability remained relatively high at 24.2%, compared to the cancer cell lines, which showed much lower viability at the same concentration. At 50 μM, HEK293 cells maintained 31.3% viability, while at 10 μM, viability was 44.1%, which is significantly higher than in the cancer cells. At the lower concentrations of 1 and 0.1 μM, viability was 57.1 and 87.3%, respectively.

TZEP7 exhibits a strong, dose-dependent cytotoxic effect on PC-3, A549, and MCF-7 cancer cells, with a significantly higher IC_50_ in normal HEK293 cells. These data suggest that TZEP7 has the potential for selective anticancer activity, with a favorable therapeutic window. The compound’s ability to induce cell death in cancer cells while sparing normal cells makes it a promising candidate for further exploration in cancer treatment, particularly in combination with other agents that could enhance its pro-apoptotic effects.

### TZEP7-Induced Apoptotic Cell Death

A chemotherapeutic approach to combat cancer involves focusing on apoptosis, a programmed cell death mechanism.^26^ Key characteristics of apoptotic cell demise include nucleus fragmentation and cellular shrinkage. This process unfolds through two main pathways: extrinsic and intrinsic, both intertwined with mitochondrial pathways. Evaluating apoptosis typically aligns with inhibiting cellular proliferation, a vital aspect of the biological reaction to various chemotherapy agents.^27^ Consequently, after establishing the IC_50_ values of TZEP7 for all cancer cells, the induction of apoptosis was evaluated using flow cytometry.

**Figure 3:**
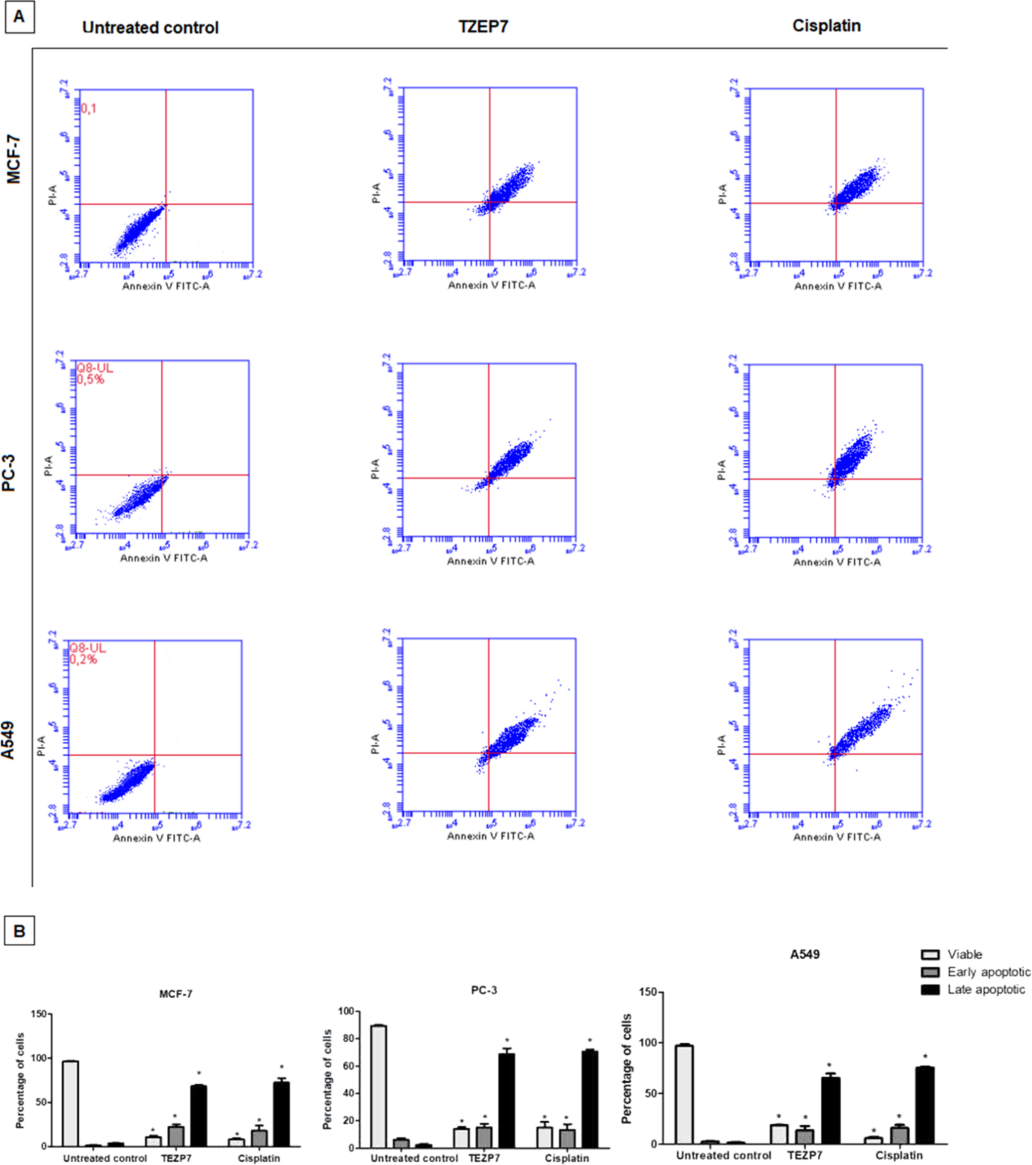
(A) Flow cytometric analysis of apoptosis in MCF-7 (human breast cancer), PC-3 (human prostate cancer), and A549 (human lung carcinoma) cells treated with TZEP7 (a novel compound) and cisplatin (a known chemotherapeutic agent) for 72 h. Cells were stained with Annexin V-FITC and PI to distinguish between live, early apoptotic, and late apoptotic cells. The untreated control groups were included to establish baseline apoptotic levels. The assay was performed to evaluate the pro-apoptotic effects of TZEP7 in comparison with cisplatin, a standard reference drug. (B) Quantitative analysis of the apoptotic populations (viable, early apoptotic, and late apoptotic) in MCF-7, PC-3, and A549 cells following treatment with TZEP7 and cisplatin for 72 h. Both TZEP7 and cisplatin significantly increased the early and late apoptotic populations compared to the untreated control (*p* < 0.05). Data are presented as mean ± SEM from three independent experiments, and statistical significance was determined using one-way ANOVA followed by Tukey’s posthoc test (*p < 0.05 vs untreated control).

Cancer cells were treated with the calculated IC_50_ values of TZEP7 and reference drug cisplatin for 72 h and subsequently analyzed. As shown in Figure 3, by cisplatin treatment, in MCF-7 breast cancer cells, 18.7% of cells were in early apoptosis, while 72.2% were in late apoptosis (*p* < 0.05). For A549 lung cancer cells, the percentages of early and late apoptotic cells were 13.4 and 70.8%, respectively (*p* < 0.05). In PC-3 prostate cancer cells, 16.2% of cells were in early apoptosis, with 75.4% in late apoptosis (*p* < 0.05). After treatment with TZEP7, 22.4% of cells were in early apoptosis, while 68.5% were in late apoptosis in MCF-7 breast cancer cells (*p* < 0.05) (Figure 3). For A549 lung cancer cells, the percentages of early and late apoptotic cells were 15.1 and 68.7%, respectively (*p* < 0.05). In PC-3 prostate cancer cells, 13.7% of cells were in early apoptosis, with 65.6% in late apoptosis (*p* < 0.05) (Figure 3). These findings indicate that TZEP7 effectively induces apoptotic cell death across various cancer types.

### Verifying Apoptosis Induction via qRT-PCR and Molecular Docking Analysis

Characterized by evading apoptosis and uncontrolled proliferation, cancer cells are a focus of anticancer drug development strategies, which predominantly target the apoptotic pathway and associated protein structures.^24^ Alterations within the mitochondria play a pivotal role in orchestrating the control of Bcl-2 family proteins and caspase-independent apoptosis. Bcl-2 and Bax, key members of the Bcl-2 family exerting contrasting effects on apoptosis, play essential roles in regulating mitochondrial membrane permeability, function, and cytochrome release.

Caspases are a family of protease enzymes that play a central role in apoptosis by executing cell death pathways. Caspase-8 and caspase-9 are key regulators of apoptosis via different pathways.^28^ Caspase-8 is primarily involved in the extrinsic apoptosis pathway, which is triggered by external signals such as death ligands binding to death receptors on the cell surface. Activation of caspase-8 leads to the activation of downstream caspases and ultimately results in apoptosis. Caspase-9, on the other hand, is a central player in the intrinsic apoptosis pathway, also known as the mitochondrial pathway. This pathway is initiated by intracellular signals such as DNA damage or cellular stress, leading to the release of cytochrome c from mitochondria. Cytochrome c binds to Apaf-1 (apoptotic protease activating factor 1), forming the apoptosome complex, activating caspase-9. Activated caspase-9 subsequently triggers a cascade of caspase activation, leading to apoptosis.^29^

**Figure 4:**
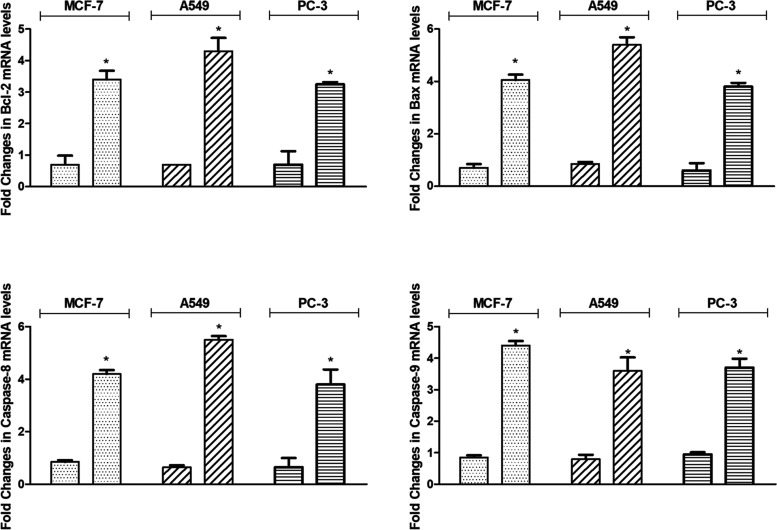
Fold changes in mRNA levels of apoptosis-related proteins (Bcl-2, Bax, Caspase-8, and Caspase-9) in MCF-7, A549, and PC-3 cells after treatment with the IC_50_ concentration of TZEP7 for 72 h. The levels of these proteins were measured by quantitative RT-PCR, normalized to GAPDH, and expressed as fold changes relative to the untreated control. Significant increases in Bax, Caspase-8, and Caspase-9 levels, alongside a decrease in Bcl-2 levels, suggest that TZEP7 induces apoptosis through the intrinsic and extrinsic pathways in these cancer cell lines (*p* < 0.05).

To confirm the induction of apoptotic cell death in cancer cells by TZEP7 treatment, mRNA levels of apoptosis-related proteins Bcl-2, Bax, caspase-8, and caspase-9 were analyzed using RT-qPCR. According to Figure 3, treatment with TZEP7 for 72 h significantly reduced antiapoptotic Bcl-2 levels by 3.4-, 3.3-, and 4.3-fold in MCF-7, A549, and PC-3 cells, respectively. Conversely, levels of pro-apoptotic Bax increased by 4.1-, 4.8-, and 4.3-fold following TZEP7 treatment in MCF-7, A549, and PC-3 cells, respectively (Figure 4). Caspase-8 levels were induced by 4.2, 3.8- and 5.4- fold and caspase-9 levels were caused by 4.4-, 3.7- and 3.6- fold in MCF-7, A549, and PC-3 cells, respectively (Figure 4).

These results underscore the activation of both intrinsic and extrinsic apoptosis by TZEP7 in all tested cancer cell lines, along with significant changes observed in essential apoptotic proteins.

### Inhibitory Impact of TZEP7 on EGFR Kinase Activity

**Figure 5:**
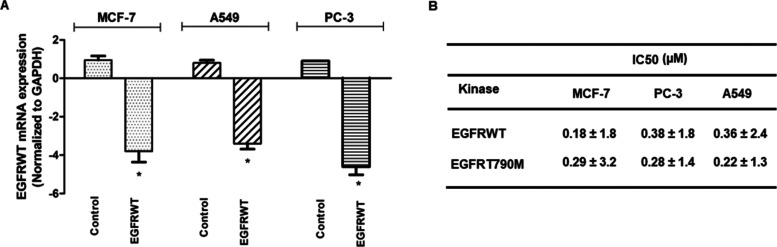
(A) Downregulation of epidermal growth factor receptor (EGFR) mRNA expression by TZEP7 in MCF-7, A549, and PC-3 cells after 72 h of treatment. The mRNA levels were normalized to GAPDH and compared to the untreated control group. Significant downregulation of EGFRWT (wild-type) expression was observed in all cell lines (*p* < 0.05). (B) Inhibition of EGFR wild-type and mutant (T790M) kinase activity by TZEP7, determined via ELISA-based kinase inhibition assays. The IC_50_ values represent the concentration of TZEP7 required to inhibit 50% of kinase activity and are presented as the mean ± SD from at least three independent experiments (*p* < 0.05).

Inhibiting cell proliferation by inactivating EGFR, which is commonly overactivated and/or mutant in cancer cells, is one of the main goals in developing new anticancer agents. Here, alongside evaluating the cytotoxic and apoptotic effects of the synthesized TZEPs, we explored their interactions with the epidermal growth factor receptor (EGFR), a significant therapeutic target in cancer treatment. This examination was conducted using a homogeneous time-resolved fluorescence (HTRF) assay. The results revealed that SPP10 exhibited EGFR kinase inhibitor activity, with IC_50_ values ranging from 0.28 to 0.38 μM across various cancer cells. In PC-3 and A549 cells, TZEP7 demonstrated comparable activity to EB, with IC_50_ values of 0.38 ± 1.8 and 0.36 ± 2.4 μM, respectively. Interestingly, TZEP7 displayed increased potency against EGFR compared to EB in MCF-7 cells, with an IC_50_ value of 0.18 ± 1.8 μM versus 0.30 ± 3.2 μM. Furthermore, TZEP7 exhibited similar inhibitory activity against EGFRT790 M kinase as EB, with IC_50_ values of 0.29 ± 3.2, 0.22 ± 1.3, and 0.28 ± 1.4 μM in MCF-7, A549, and PC-3 cells, respectively. Inhibition of EGFR kinase activity was also verified via RT-qPCR. As shown in Figure 5, by TZEP7 treatment EGFRWT was downregulated by 3.8-, 4.6-, and 3.4-fold in MCF-7, A549, and PC-3 cells, respectively (*p* < 0.05).

### Molecular Docking TZEP7 with EGFRMT and EGFRT790M

In human cancer, EGFR undergoes frequent modifications such as overexpression, amplification, and mutation, rendering it one of the most frequently altered genes. Targeting EGFR activity specifically disrupts signal transduction pathways that control tumor cell growth, proliferation, and resistance to apoptosis. Clinical therapies for numerous malignancies commonly employ small-molecule tyrosine kinase inhibitors and monoclonal antibodies, both widely acknowledged as typical agents directed against EGFR.^4^

**Figure 6:**
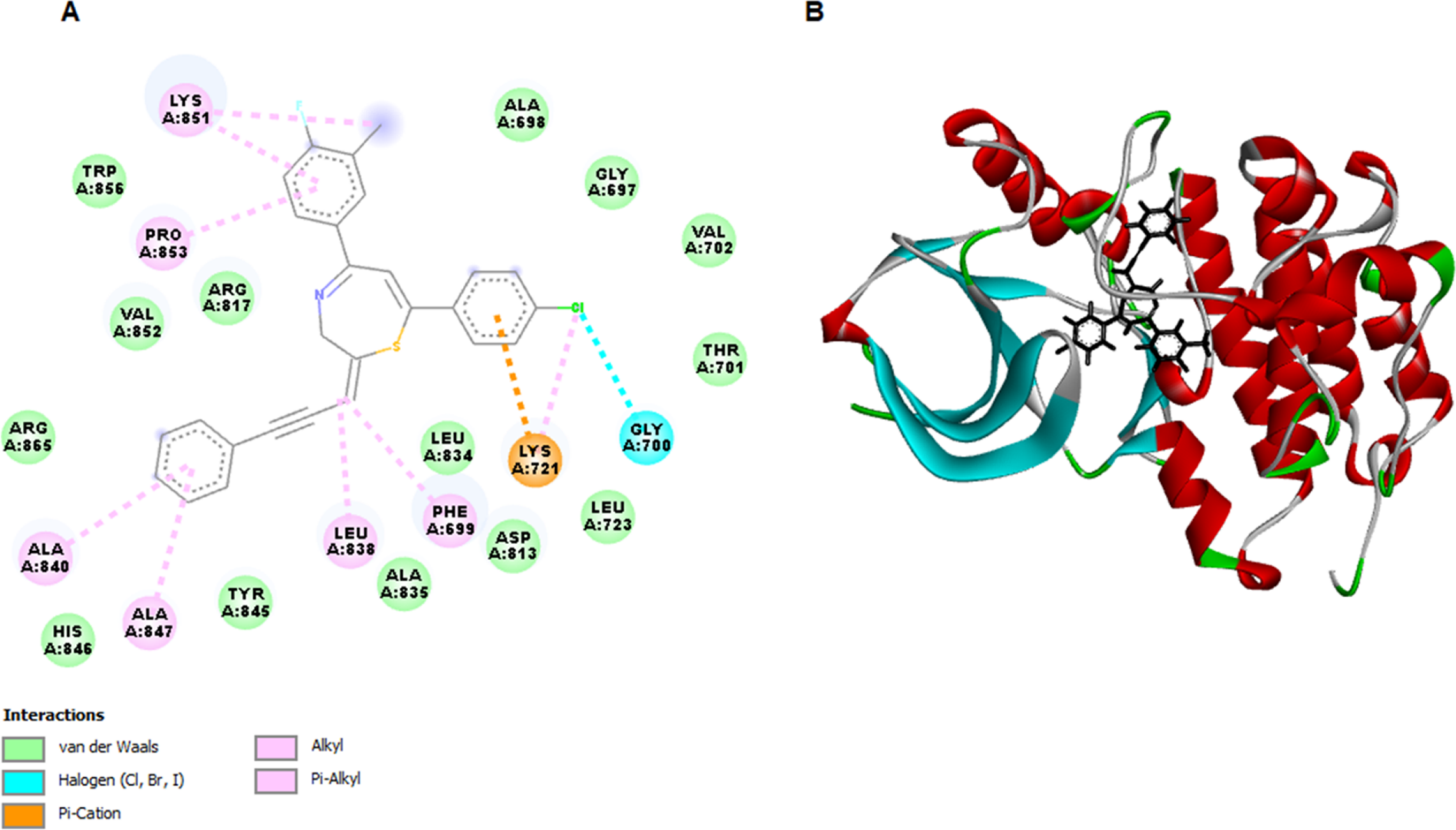
Molecular docking analysis of TZEP7 with epidermal growth factor receptor wild-type (EGFRWT). (A) 2D interaction map showing the specific hydrogen bonds, hydrophobic interactions, and other key interactions between TZEP7 and the active site residues of EGFRWT. (B) 3D interaction map illustrating the spatial orientation of TZEP7 within the EGFRWT binding pocket, highlighting the critical amino acid residues involved in binding. This analysis was conducted to explore the potential binding affinity and interaction mechanisms of TZEP7 with EGFRWT, which could be relevant for its inhibitory activity.

**Figure 7:**
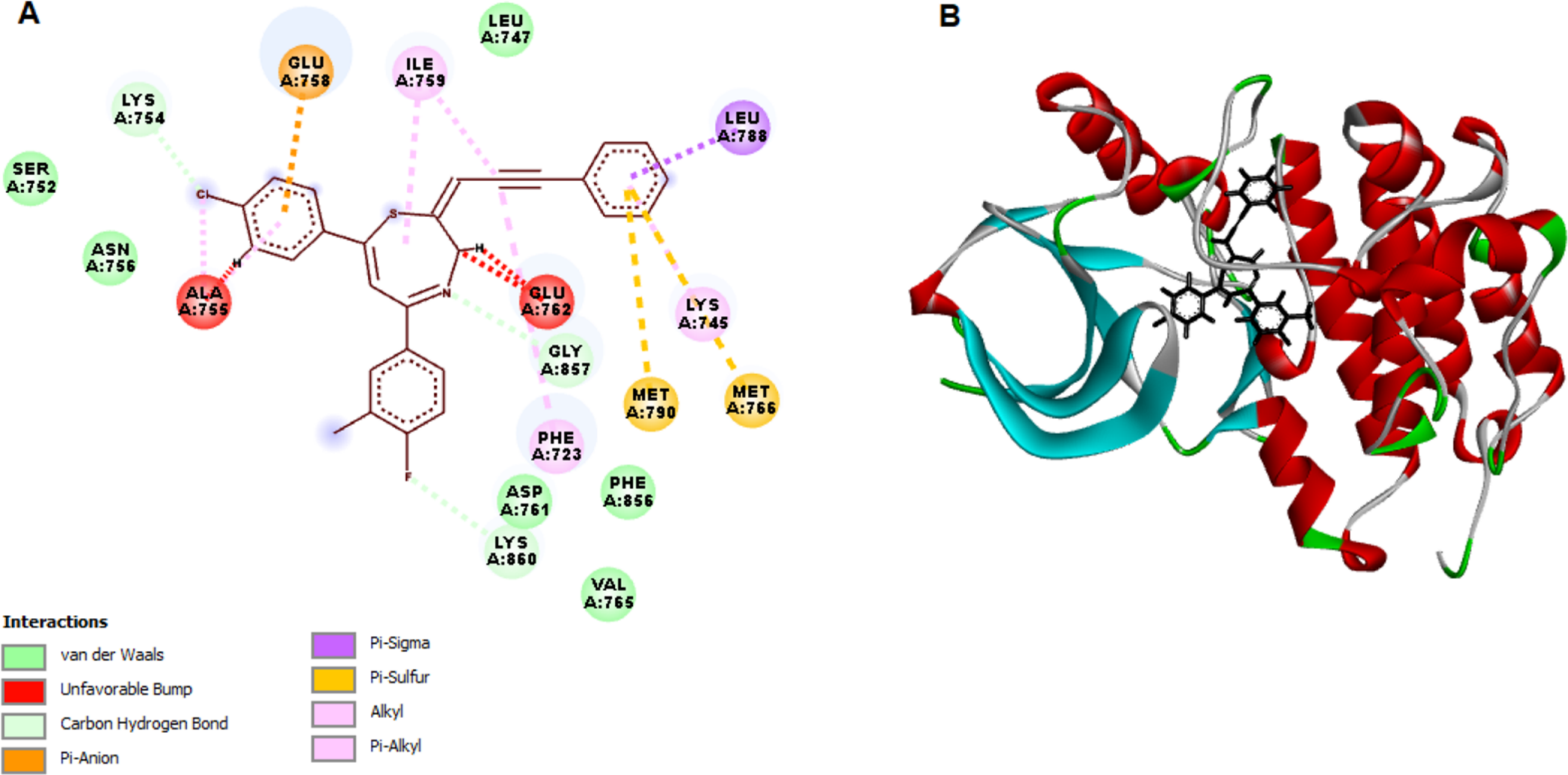
Molecular docking analysis of TZEP7 with epidermal growth factor receptor T790 M mutant (EGFRT790 M). (A) 2D interaction map illustrating the hydrogen bonds, hydrophobic interactions, and other key interactions between TZEP7 and the active site residues of the EGFRT790 M mutant. (B) 3D interaction map depicting the spatial arrangement of TZEP7 within the EGFRT790 M binding pocket, emphasizing the critical residues involved in binding. This docking analysis was performed to investigate the binding affinity and interaction mechanisms of TZEP7 with the EGFRT790 M mutation, which is often associated with resistance to EGFR inhibitors in cancer therapy.

Following the observed EGFR inhibition, in silico molecular docking studies were conducted to analyze the characteristics, binding energy, and stability of the interactions. Docking studies were conducted against two variants of EGFRWT and the mutant type, EGFRT790 M (Figures 6 and 7). The validation of TZEP7 was conducted based on their binding affinities with the respective receptor targets. Table 4 presents the docking scores (in kcal/mol), hydrogen bond interactions, bond lengths, and the amino acids involved in these interactions. The binding energy of TZEP7 closely resembled that of the native EGFR ligand EB, consistent with the findings of the EGFR kinase inhibitory assay. TZEP7 exhibited a strong binding energy of −153.424 kcal/mol, indicating robust interactions with EGFRWT. TZEP7 interacted with the binding site of EGFRWT via alkyl and π–alkyl interactions with LYS721, LYS851, PRO853, LEU838, and ALA840. Other steric interactions were formed via GLY700 and PHE699 (Figure 6). Molecular docking analysis was also performed to determine the interaction level of TZEP7 with the EGFRT790 M mutant. As shown in Table 4, TZEP7 showed strong binding affinity (−129.858 kcal/mol) with mutant EGFRT790M. TZEP7 formed hydrogen bonds with LYS754, LYS860, and GLY857 and steric interactions (alkyl, pi-alkyl, pi-sigma, pi-anion and pi-sulfur) with GLU758, ILE759, LEU788, MET766, MET790, GLU762, PHE723 (Figure 7).

**Table 4:** Molecular Docking Analysis Was Employed to Evaluate the Interaction between TZEP7, EGFRWT, and EGFRT790M

	EGFRWT			EGFRT790M		
	docking score (kcal/mol)	H bond interaction	other interaction	docking score (kcal/mol)	H bond interaction	other interaction
TZEP7	–153.424		GLY700, LEU838, LYS721, LYS851, PRO853, LEU838, ALA840, ALA847, PHE699	–129.858	LYS754, LYS860, GLY857	GLU758, ILE759, LEU788, MET766, MET790, GLU762, PHE723
EB	–179.941	ALA698, PHE699, ARG817, ASN818, TYR867	PHE699, LEU838, ALA835, ALA 840.	–150.700	LYS745	MET790, MET766, LEU844, LEU718, LEU792

The inhibition of EGFR by 1,4-thiazepine derivatives involves binding to the kinase domain, preventing ATP from accessing the binding site. This blockade hampers the receptor’s autophosphorylation and subsequent activation of downstream signaling cascades ultimately leading to reduced cancer cell proliferation and increased apoptosis.

### AdmetSAR Analysis of TZEP7

The AdmetSAR analysis aids researchers in optimizing the potency, selectivity, and pharmacokinetic properties of lead compounds. By understanding the structure–activity relationship, researchers can design and synthesize analogs with improved efficacy and reduced toxicity.

The AdmetSAR 2.0 online tool (http://lmmd.ecust.edu.cn/admetsar2) was employed to predict the absorption, distribution, metabolism, excretion, and toxicity (ADMET) properties of the TZEP7 compound. This platform provides detailed information on several physicochemical parameters, such as molecular weight (M.W.), log *P*_o/w_ (octanol–water partition coefficient), log *S* (solubility), log *K*_p_ (skin permeation), hydrogen bond acceptors (HBA), hydrogen bond donors (HBD), total polar surface area (TPSA), and molar refractivity (MR). These parameters are crucial for understanding the ADMET characteristics of any drug or organic molecule.

According to ADMET predictions, drug candidates should conform to the Rule of Five (Ro5) with no more than one violation. In our analysis, TZEP7 demonstrated a molecular weight of 443.090 and contained 1 hydrogen bond acceptor and 0 hydrogen bond donors, thereby adhering to Ro5 guidelines. The logarithm of the *n*-octanol/water partition coefficient ( log *P*_o/w_) is a measure of lipophilicity, which is critical for transport mechanisms such as membrane permeability. TZEP7 exhibited a log *P*_o/w_ value of 4.81, suggesting moderate permeability. Total polar surface area (TPSA), representing the sum of polar atoms primarily oxygen and nitrogen, is an indicator of permeability, with higher values generally implying lower permeability. TZEP7 had a TPSA value of 12.360, indicating potential for good permeability. TPSA was utilized to estimate the percentage of absorption (%ABS), which was calculated to be 99% for TZEP7, reflecting excellent cellular membrane permeability. These results confirm that TZEP7 meets Ro5 criteria: molecular weight ≤500 Da, log *P* < 5, *n*HBD ≤ 5, *n*HBA ≤ 10, and TPSA < 140 Å^2^. Furthermore, the logS value for TZEP7 was −6.452, indicating moderate water solubility. The human intestinal absorption value was 0.004, with bioavailability values of 20 and 30% was 0.002 and 0.001, respectively, signifying good bioavailability. AdmetSAR analysis indicated favorable lipophilicity, a low fraction unbound, and appropriate dispersion, suggesting high bioavailability for TZEP7. The enyne moiety can influence the metabolic stability of the molecule. Enynes can undergo metabolic transformations, such as oxidation and reduction, which can either stabilize the drug or lead to the formation of active or inactive metabolites. The presence of conjugated double and triple bonds can also make the molecule less susceptible to rapid degradation by metabolic enzymes. Enhanced metabolic stability can lead to a slower excretion rate, prolonging the drug’s presence in the system. Conversely, metabolites of enyne-containing compounds may be more readily excreted via renal or hepatic pathways.

## Conclusions

The results of this study highlight the potential of 1,4-thiazepine compounds having enyne moiety, with TZEP7 emerging as an especially promising candidate. TZEP7 demonstrates significant cytotoxicity in breast, lung, and prostate cancer cells, and its selective effect on cancer cells underscores its promise as an effective anticancer agent. The ability of TZEP7 to induce apoptosis, indicated by changes in key apoptotic proteins, enhances its therapeutic appeal. Additionally, the compound shows noteworthy EGFR kinase inhibitory activity, underscoring its potential as a targeted cancer therapy. Comprehensive in vitro evaluations including assessments of cytotoxicity, apoptosis induction, and molecular interactions position TZEP7 as a strong candidate for further cancer treatment research. This study not only provides valuable insight into the development of new anticancer agents, but also highlights the importance of novel synthesized 4-thiazepine compounds in advancing cancer research and treatment strategies.

## Supporting Information Available

Experimental procedures, spectroscopic data, and copies of ^1^H and ^13^C NMR spectra for all 1,4-thiazepine (TZEP) derivatives (PDF)
