# Supplementary material for: Novel Enyne-Modified 1,4-Thiazepines as Epidermal Growth Factor Receptor Inhibitors: Anticancer and Computational Studies
Source: ACS Omega. 2024 Dec 26;10(1):821–32. doi: 10.1021/acsomega.4c07877 (PMC11740379; doi:10.1021/acsomega.4c07877)
Supplement: Supplementary file 1 — ao4c07877_si_001.pdf [file ao4c07877_si_001.pdf]

SUPPORTING INFORMATION

for

Novel Enyne-Modified 1,4-Thiazepines as Epidermal Growth Factor Receptor Inhibitors: Anticancer and Computational Studies

Harika Atmaca,[1] Çisil Çamlı Pulat,[2] Suleyman Ilhan,[1] Elif Serel Yilmaz,[3] Metin Zora[3,*]

[1]*Department of Biology, Faculty of Engineering and Natural Sciences, Manisa Celal Bayar University, 45140 Manisa, Turkey*

[2]*Applied Science Research Center, Manisa Celal Bayar University, 45140 Manisa, Turkey*

[3]*Department of Chemistry, Middle East Technical University, 06800 Ankara, Turkey*

Table of Contents

Experimental Section ……………………………………………… S2-S8

Copies of ^{1}H and ^{13}C NMR Spectra ……………………………… S9-S20

References ………………………………………………………… S21

* Corresponding Author. Email: zora@metu.edu.tr (M.Z.)

Experimental Section

General information. ^{1}H and ^{13}C NMR spectra were recorded at 400 and 100 MHz, respectively. Chemical shifts were given in parts per million (ppm) relative to $CDCl_3$ (7.26 and 77.16 ppm in ^{1}H and ^{13}C NMR, respectively). Coupling constants (J) were given in Hertz (Hz), and spin multiplicities were shown by the following symbols: s (singlet), d (doublet), t (triplet), q (quartet), m (multiplet). DEPT ^{13}C NMR information is depicted in parentheses as C, CH, CH_2 and CH_3. Infrared spectra (IR) were obtained by using attenuated total reflection (ATR). Band positions diagnostic for major functional groups were recorded in reciprocal centimeters (cm^{-1}). Mass spectra (MS) and high resolution mass spectra (HRMS) were obtained by using Electrospray Ionization (ESI) with Micro-Tof; m/z values are reported (For each measurement, the mass scale was recalibrated with sodium formate clusters, and samples were dissolved and measured in MeOH or CH_3CN). Melting points were determined on an automated instrument. Flash chromatography was performed using thick-walled glass columns and “flash grade” silica gel (230-400 mesh). Thin layer chromatography (TLC) was accomplished by using commercially prepared 0.25 mm silica gel and visualization was effected with short wavelength UV lamp (254 nm). The relative proportions of solvents in chromatography solvent mixtures refer to the volume:volume ratio. All commercially available reagents were used directly without purification unless otherwise stated. All solvents used in reactions and chromatography were distilled and/or dried properly for purity. The inert atmosphere was created by slight positive pressure (ca. 0.1 psi) of argon. All glassware was dried in oven prior to use.

N-(5-Phenyl-2,4-pentadiynyl) β-enaminones (**NPEs**) were prepared according to our previous studies.[1,2] 2-(2-Propyn-1-ylidene)-2,3-dihydro-1,4-thiazepines (**TZEPs**) were synthesized according to our previous study as depicted in Table S1.[2]

Table S1. Scope of the Synthesis of 1,4-Thiazepines (TZEPs)[a]

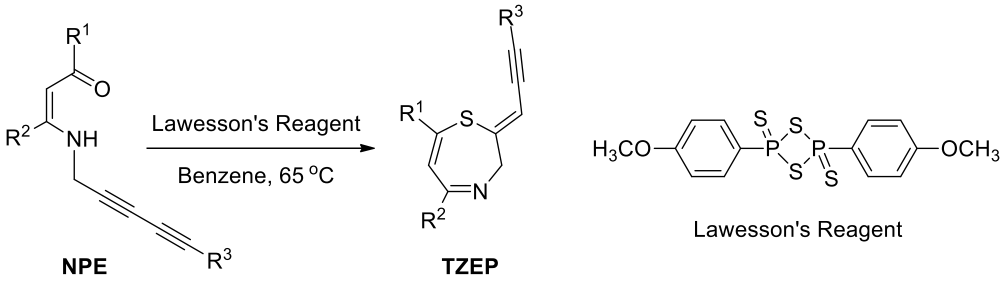

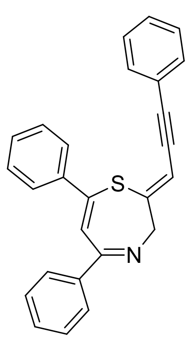

TZEP1 (64%)

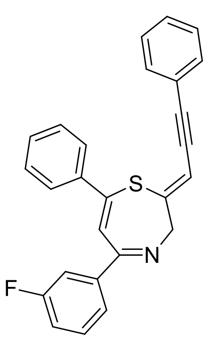

TZEP2 (57%)

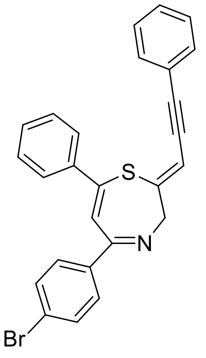

TZEP3 (31%)

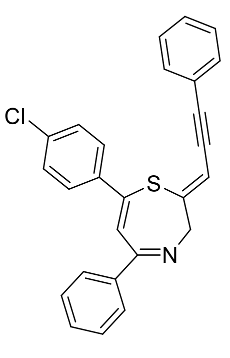

TZEP4 (23%)

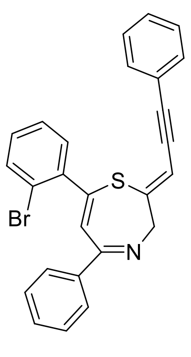

TZEP5 (46%)

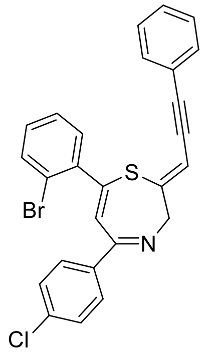

TZEP6 (46%)

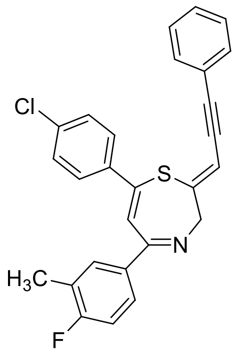

TZEP7 (49%)

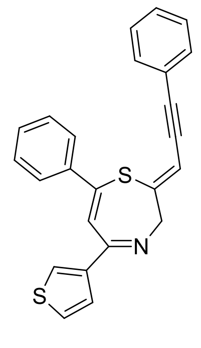

TZEP8 (15%)

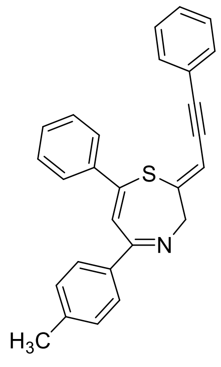

TZEP9 (25%)

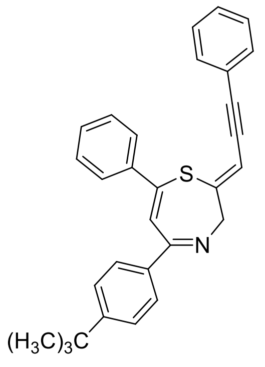

TZEP10 (36%)

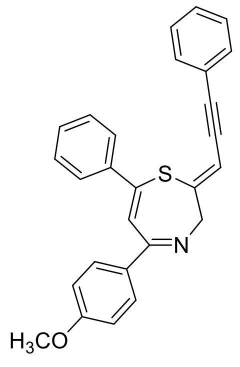

TZEP11 (43%)

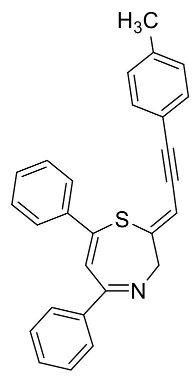

TZEP12 (30%)

[a]Isolated yields.

General Procedure for the synthesis of 2-(2-propyn-1-ylidene)-2,3-dihydro-1,4-thiazepines (TZEPs). To a stirred solution of *N*-(2,4-pentadiynyl)-β-enaminone (**NPE**) (0.25 mmol) in benzene (5.0 mL) under argon was added Lawesson's reagent (0.25 mmol). The resulting mixture was then heated at 65 °C for approximately 20 min to 3.5 h. Note that reaction was continued until *N*-(2,4-pentadiynyl)-β-enaminone (**NPE**) was completely consumed as monitored by routine TLC analysis. After the reaction was over, the solvent was removed on a rotary evaporator, and ethyl acetate (40 mL) and a saturated aqueous solution of NH_4Cl (15 mL) were added. After the layers were separated, the aqueous layer was extracted with ethyl acetate (2 x 30 mL). The combined organic layers were dried over $MgSO_4$ and evaporated on a rotary evaporator to give the crude product, which was purified by flash chromatography on silica gel using 9:1 hexane/ethyl acetate as the eluent to afford the corresponding 1,4-thiazepine (**TZEP**) derivative with the indicated yield shown in Table S1.

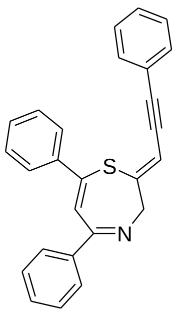

(*Z*)-5,7-Diphenyl-2-(3-phenylprop-2-yn-1-ylidene)-2,3-dihydro-1,4-thiazepine (TZEP1). An orange-brown solid (R_f = 0.53 in 4:1 hexane/ethyl acetate; mp 94.0-95.0 °C). ^{1}H NMR (400 MHz, $CDCl_3$) δ 7.82 (dd, *J* = 7.6, 1.8 Hz, 2H), 7.75 (dd, *J* = 6.8, 2.8 Hz, 2H), 7.50-7.40 (m, 8H), 7.34-7.21 (m, 3H), 6.95 (s, 1H), 5.80 (s, 1H), 4.88 (s, 2H); ^{13}C NMR (100 MHz, $CDCl_3$) δ 168.0 (C), 150.6 (C), 149.8 (C), 140.0 (C), 138.9 (C), 131.5 (CH), 130.7 (CH), 130.2 (CH), 128.9 (CH), 128.6 (CH), 128.4 (CH), 128.0 (CH), 127.8 (CH), 124.0 (C), 123.3 (CH), 103.7 (CH), 98.6 (C), 85.3 (C), 58.6 (CH_2) (Note that two CH peaks overlap on each other); IR (neat): 3021, 2917, 2849, 2240, 2051, 1607, 1569, 1486, 1323, 1293, 1246, 1174, 1158, 1112, 1067, 1025, 935, 915, 839, 814, 779, 755, 689, 663, 620, 572, 562, 526, 417 cm^{-1}; MS (ESI, *m/z*): 376.11 [M-H]$^-$; HRMS (ESI) calcd. for $C_{26}H_{18}NS$: 376.1165 [M-H]$^-$, found: 376.1174.

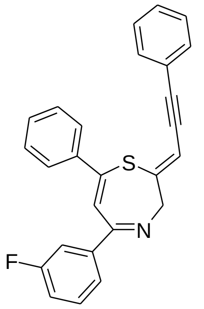

(*Z*)-5-(3-Fluorophenyl)-7-phenyl-2-(3-phenylprop-2-yn-1-ylidene)-2,3-dihydro-1,4-thiazepine (TZEP2). A reddish brown solid (R_f = 0.58 in 4:1 hexane/ethyl acetate; mp 44.1-45.1 °C). ^{1}H NMR (400 MHz, $CDCl_3$) δ 7.62 (dd, *J* = 6.2, 2.9 Hz, 2H), 7.52-7.41 (m, 2H), 7.38–7.28 (m, 6H), 7.20-7.14 (m, 3H), 7.04 (td, *J* = 8.3, 2.5 Hz, 1H), 6.78 (s, 1H), 5.68 (s, 1H), 4.77 (s, 2H); ^{13}C NMR (100 MHz, $CDCl_3$) δ 166.8 (d, ^4J = 2.5 Hz, C), 163.0 (d, ^1J = 246.5 Hz, CF), 150.5 (C), 150.1 (C), 141.1 (d, ^3J = 7.2 Hz, C), 139.8 (C), 131.5 (CH), 130.3 (CH), 130.1 (d, ^3J = 8.1 Hz, CH) ,129.0 (CH), 128.5 (CH), 128.4 (CH), 127.9 (CH), 123.5 (d, ^4J = 2.7 Hz, CH) 123.3 (CH), 123.2 (C), 117.5 (d, ^2J = 21.4 Hz, CH), 114.7 (d, ^2J = 22.6 Hz,

CH), 104.1 (CH), 98.8 (C), 85.2 (C), 58.6 (CH_2); IR (neat): 3026, 2961, 2849, 2192, 1733, 1577, 1485, 1440, 1296, 1259, 1172, 1023, 937, 875, 789, 754, 686, 616, 568, 524, 480 cm^{-1}; MS (ESI, *m/z*): 394.10 $[M+H]^+$; HRMS (ESI) calcd. for $C_{26}H_{19}FNS$: 394.1071 $[M+H]^+$, found: 394.1078.

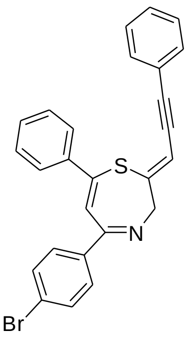

(*Z*)-5-(4-Bromophenyl)-7-phenyl-2-(3-phenylprop-2-yn-1-ylidene)-2,3-dihydro-1,4-thiazepine (TZEP3). An orange-yellow solid (R_f = 0.60 in 4:1 hexane/ethyl acetate; mp 98.1-98.7 °C). ^1H NMR (400 MHz, $CDCl_3$) δ 7.76-7.63 (m, 4H), 7.56 (d, *J* = 8.6 Hz, 2H), 7.48-7.39 (m, 5H), 7.31-7.24 (m, 3H), 6.88 (s, 1H), 5.78 (s, 1H), 4.86 (s, 2H); ^{13}C NMR (100 MHz, $CDCl_3$) δ 166.8 (C), 150.3 (C), 150.2 (C), 139.9 (C), 137.9 (C), 131.8 (CH), 131.5 (CH), 130.3 (CH), 129.3 (CH), 129.0 (CH), 128.5 (CH), 128.4 (CH), 127.9 (CH), 125.1 (CBr), 123.4 (C), 123.2 (CH), 103.8 (CH), 98.7 (C), 85.2 (C), 58.8 (CH_2); IR (neat): 3020, 2923, 2868, 2349, 2138, 2029, 1742, 1606, 1485, 1441, 1392, 1325, 1295, 1254, 1174, 1069, 1007, 934, 918, 818, 757, 690, 526, 481, 422 cm^{-1}; MS (ESI, *m/z*): 456.04 $[M+H]^+$; HRMS (ESI) calcd. for $C_{26}H_{19}BrNS$: 456.0416 $[M+H]^+$, found: 456.0416.

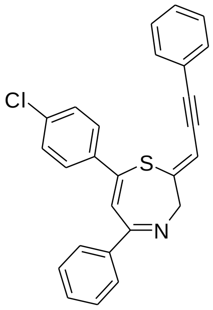

(*Z*)-7-(4-Chlorophenyl)-5-phenyl-2-(3-phenylprop-2-yn-1-ylidene)-2,3-dihydro-1,4-thiazepine (TZEP4). An orange-brown solid (R_f = 0.50 in 4:1 hexane/ethyl acetate; mp 68.1-69.2 °C). ^1H NMR (400 MHz, $CDCl_3$) δ 7.79 (d, *J* = 8.1 Hz, 2H), 7.68 (d, *J* = 8.3 Hz, 2H), 7.50-7.29 (m, 7H), 7.32–7.27 (m, 3H), 6.92 (s, 1H), 5.79 (s, 1H), 4.86 (s, 2H); ^{13}C NMR (100 MHz, $CDCl_3$) δ 167.9 (C), 153.8 (C), 150.3 (C), 138.8 (C), 138.4 (CCl), 136.3 (C), 131.6 (CH), 130.7 (CH), 129.3 (CH), 129.2 (CH), 128.7 (CH), 128.5 (CH), 128.4 (CH), 127.8 (CH), 124.4 (C), 123.2 (CH), 104.0 (CH), 98.8 (C), 85.2 (C), 58.5 (CH_2); IR (neat): 3055, 3019, 2930, 2849, 2012, 1973, 1734, 1655, 1569, 1484, 1441, 1398, 1294, 1253, 1175, 1088, 1011, 905, 823, 753, 727, 687, 524, 468, 407 cm^{-1}; MS (ESI, *m/z*): 412.09 $[M+H]^+$; HRMS (ESI) calcd. for $C_{26}H_{19}ClNS$: 412.0921 $[M+H]^+$, found: 412.0932.

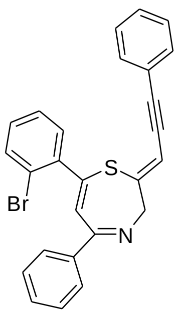

(*Z*)-7-(2-Bromophenyl)-5-phenyl-2-(3-phenylprop-2-yn-1-ylidene)-2,3-dihydro-1,4-thiazepine (TZEP5). An orange solid (R_f = 0.52 in 4:1 hexane/ethyl acetate; mp 68.1-69.0 °C). ^1H NMR (400 MHz, $CDCl_3$) δ 7.78 (dd, *J* = 7.8, 1.6 Hz, 2H), 7.64 (dd, *J* = 8.0, 0.8 Hz, 1H), 7.48-7.33 (m, 7H), 7.29-7.18 (m, 4H), 6.72 (s, 1H), 5.76 (s, 1H), 4.96 (s, 2H); ^{13}C NMR (100 MHz, $CDCl_3$) δ 167.1 (C), 151.4 (C), 148.8 (C), 140.8 (C), 138.8 (C), 133.5

(CH), 131.5 (CH), 130.9 (CH), 130.6 (CH), 128.6 (CH), 128.40 (CH), 128.36 (CH), 127.8 (CH), 127.7 (CH), 127.4 (CBr), 123.2 (C), 122.7 (CH), 103.5 (CH), 98.6 (C), 85.2 (C), 58.7 (CH_2) (Note that two CH peaks overlap on each other); IR (neat): 3026, 2923, 2850, 2160, 1721, 1609, 1595, 1570, 1486, 1461, 1441, 1323, 1294, 1244, 1114, 1024, 933, 817, 751, 686, 584, 525, 439 cm^{-1}; MS (ESI, *m/z*): 454.02 $[M-H]^-$; HRMS (ESI) calcd. for $C_{26}H_{17}BrNS$: 454.0271 $[M-H]^-$, found: 454.0259.

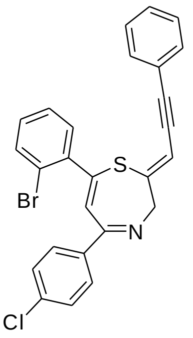

(*Z*)-7-(2-Bromophenyl)-5-(4-chlorophenyl)-2-(3-phenylprop-2-yn-1-ylidene)-2,3-dihydro-1,4-thiazepine (TZEP6). A reddish orange solid (R_f = 0.55 in 4:1 hexane/ethyl acetate; mp 87.2-88.1 °C). ^1H NMR (400 MHz, $CDCl_3$) δ 7.65 (dt, *J* = 4.3, 2.4 Hz, 3H), 7.57 (d, *J* = 7.8 Hz, 2H), 7.36 (dd, *J* = 7.6, 1.7 Hz, 2H), 7.33-7.24 (m, 3H), 7.22-7.12 (m, 3H), 6.58 (s, 1H), 5.68 (s, 1H), 4.87 (s, 2H); ^{13}C NMR (100 MHz, $CDCl_3$) δ 166.0 (C), 150.8 (C), 149.6 (C), 140.6 (C), 137.1 (C), 136.8 (CCl), 133.5 (CH), 131.5 (CH), 130.9 (CH), 130.7 (CH), 129.1 (CH), 128.8 (CH), 128.5 (CH), 128.4 (CH), 127.7 (CH), 126.7 (CBr), 123.1 (C), 122.6 (CH), 104.0 (CH), 98.8 (C), 85.1 (C), 58.6 (CH_2); IR (neat): 3053, 2926, 2848, 2201, 1732, 1610, 1590, 1486, 1462, 1398, 1370, 1239, 1174, 1089, 1045, 1011, 932, 829, 754, 733, 688, 644, 527, 502, 478, 444 cm^{-1}; MS (ESI, *m/z*): 487.98 $[M-H]^-$; HRMS (ESI) calcd. for $C_{26}H_{16}BrClNS$: 487.9881 $[M-H]^-$, found: 487.9881.

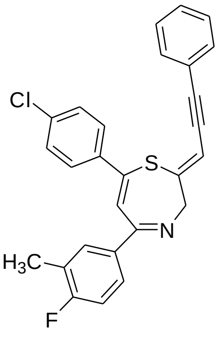

(*Z*)-7-(4-Chlorophenyl)-5-(4-fluoro-3-methylphenyl)-2-(3-phenylprop-2-yn-1-ylidene)-2,3-dihydro-1,4-thiazepine (TZEP7). An orange-brown solid (R_f = 0.66 in 4:1 hexane/ethyl acetate; mp 79.8-80.1 °C). ^1H NMR (400 MHz, $CDCl_3$) δ 7.66 (d, *J* = 8.5 Hz, 3H), 7.59-7.51 (m, 1H), 7.46-7.38 (m, 4H), 7.33-7.25 (m, 3H), 7.04 (t, *J* = 8.9 Hz, 1H), 6.87 (s, 1H), 5.78 (s, 1H), 4.83 (s, 2H), 2.32 (s, 3H); ^{13}C NMR (100 MHz, $CDCl_3$) δ 166.8 (C), 163.0 (d, ^1J = 249.4 Hz, CF), 150.4 (C), 148.4 (C), 138.4 (CCl), 136.3 (C), 134.7 (d, ^4J = 2.72 Hz, C), 131.5 (CH), 131.0 (d, ^3J = 5.8 Hz, CH), 129.22 (CH), 129.16 (CH), 128.5 (CH), 128.4 (CH), 127.1 (d, ^3J = 8.8 Hz, CH), 125.3 (d, ^2J = 17.7 Hz, C), 124.3 (C), 123.2 (CH), 115.0 (d, ^2J = 22.7 Hz, CH), 103.8 (CH), 98.8 (C), 85.1 (C), 58.5 (CH_2), 14.8 (d, ^3J = 3.4 Hz, CH_3); IR (neat): 3023, 2922, 2848, 2196, 2016, 1702, 1587, 1484, 1441, 1397, 1322, 1296, 1251, 1158, 1113, 1090, 1011, 933, 908, 813, 751, 686, 632, 591, 565, 525, 472, 452 cm^{-1}; MS (ESI, *m/z*): 442.08 $[M-H]^-$; HRMS (ESI) calcd. for $C_{27}H_{18}ClFNS$: 442.0838 $[M-H]^-$, found: 442.0855.

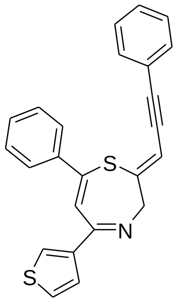

(*Z*)-7-Phenyl-2-(3-phenylprop-2-yn-1-ylidene)-5-(thiophen-3-yl)-2,3-dihydro-1,4-thiazepine (TZEP8). An orange-brown solid (R_f = 0.50 in 4:1 hexane/ethyl acetate; mp: 95.0-96.0 °C). ^{1}H NMR (400 MHz, CDCl3) δ 7.73-7.65 (m, 2H), 7.58 (dd, *J* = 5.1, 1.1 Hz, 1H), 7.48-7.40 (m, 5H), 7.34 (dd, *J* = 5.1, 2.9 Hz, 2H), 7.30-7.27 (m, 3H), 6.96 (s, 1H), 5.82 (s, 1H), 4.85 (s, 2H); ^{13}C NMR (100 MHz, $CDCl_3$), δ 163.4 (C), 154.2 (C), 149.9 (C), 139.9 (C), 131.6 (CH), 130.3 (C), 129.0 (CH), 128.5 (CH), 128.4 (CH), 128.0 (CH), 127.0 (CH), 126.4 (CH), 123.2 (C), 122.98 (CH), 104.5 (CH), 98.8 (C), 85.2 (C), 57.8 (CH_2) (Note that three CH peaks overlap on each other); IR (neat): 3020, 2916, 2847, 2189, 1593, 1565, 1486, 1441, 1414, 1294, 1247, 1176, 1081, 1023, 935, 909, 865, 840, 792, 754, 687, 614, 598, 526, 430 cm^{-1}; MS (ESI, *m/z*): 382.07 [M-H]$^{-}$; HRMS (ESI) calcd. for $C_{24}H_{16}NS_2$: 382.0730 [M-H]$^{-}$, found: 382.0718.

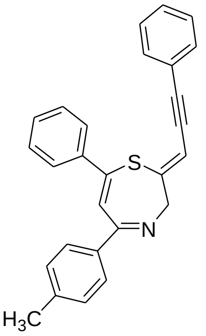

(*Z*)-7-Phenyl-2-(3-phenylprop-2-yn-1-ylidene)-5-(*p*-tolyl)-2,3-dihydro-1,4-thiazepine (TZEP9). A dark brown solid (R_f = 0.56 in 4:1 hexane/ethyl acetate; mp 78.8-79.5 °C). ^{1}H NMR (400 MHz, $CDCl_3$) δ 7.83-7.68 (m, 4H), 7.56-7.41 (m, 6H), 7.37-7.20 (m, 4H), 6.98 (s, 1H), 5.82 (s, 1H), 4.89 (s, 2H), 2.43 (s, 3H); ^{13}C NMR (100 MHz, $CDCl_3$) δ 167.9 (C), 150.7 (C), 149.7 (C), 141.0 (C), 140.1 (C), 135.97 (C), 131.5 (CH), 130.2 (CH), 129.3 (CH), 128.9 (CH), 128.4 (CH), 128.0 (CH), 127.8 (CH), 124.1 (C), 123.3 (CH), 103.7 (CH), 98.6 (C), 85.3 (C), 58.3 (CH_2), 21.6 (CH_3) (Note that two CH peaks overlap on each other); IR (neat): 3025, 2993, 2846, 2341, 2180, 2161, 2027, 1979, 1737, 1600, 1564, 1486, 1441, 1293, 1249, 1179, 1112, 1082, 1018, 933, 855, 823, 802, 762, 750, 683, 660, 568, 527, 503, 476, 435, 422 cm^{-1}; MS (ESI, *m/z*): 390.13 [M-H]; HRMS (ESI) calcd. for $C_{27}H_{20}NS$: 390.1322 [M-H]$^{-}$, found: 390.1307.

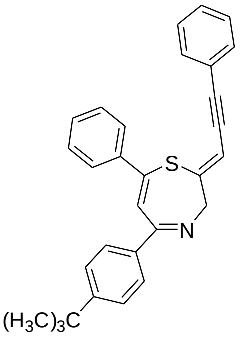

(*Z*)-5-[4-(*tert*-Butyl)phenyl]-7-phenyl-2-(3-phenylprop-2-yn-1-ylidene)-2,3-dihydro-1,4-thiazepine (TZEP10). A reddish brown solid (R_f = 0.61 in 4:1 hexane/ethyl acetate; mp 76.9-77.9 °C). ^{1}H NMR (400 MHz, $CDCl_3$) δ 7.78-7.70 (m, 4H), 7.48-7.42 (m, 7H), 7.32-7.27 (m, 3H), 6.97 (s, 1H), 5.79 (s, 1H), 4.87 (s, 2H), 1.35 (s, 9H); ^{13}C NMR (100 MHz, $CDCl_3$), δ 167.7 (C), 154.0 (C), 150.9 (C), 149.3 (C), 140.1 (C), 136.1 (C), 131.5 (CH), 130.1 (CH), 128.9 (CH), 128.4 (CH), 128.0 (CH), 127.6 (CH), 125.6 (CH), 124.3 (C), 123.3 (CH), 103.4 (CH), 98.5 (C), 85.4 (C), 58.5 (CH_2), 35.0 (C), 31.4 (CH_3) (Note that two CH peaks overlap on each other); IR (neat): 2950, 2862,

2239, 2168, 2018, 1897, 1733, 1599, 1559, 1507, 1487, 1457, 1442, 1362, 1294, 1257, 1187, 1110, 1071, 1022, 934, 827, 753, 688, 553, 526, 468 cm^{-1}; MS (ESI, *m/z*): 434.19 [M+H]$^+$; HRMS (ESI) calcd. for $C_{30}H_{28}NS$: 434.1937 [M+H]$^+$, found: 434.1952.

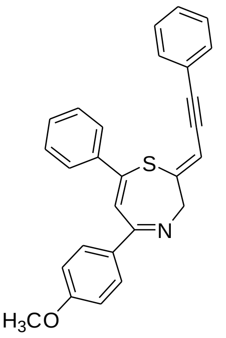

(*Z*)-5-(4-Methoxyphenyl)-7-phenyl-2-(3-phenylprop-2-yn-1-ylidene)-2,3-dihydro-1,4-thiazepine (TZEP11). An orange-yellow solid (R_f = 0.37 in 4:1 hexane/ethyl acetate; mp 58.0-59.0 °C). ^{1}H NMR (400 MHz, $CDCl_3$) δ 7.78 (d, *J* = 8.9 Hz, 2H), 7.75-7.71 (m, 2H), 7.46-7.41 (m, 3H), 7.37 (d, *J* = 4.6 Hz, 1H), 7.29-7.26 (m, 3H), 6.96-6.92 (m, 3H), 5.78 (s, 1H), 4.83 (s, 2H), 4.70 (s, 1H), 3.85 (s, 3H); ^{13}C NMR (100 MHz, $CDCl_3$) δ 167.2 (C), 161.7 (C), 151.1 (C), 149.4 (C), 140.1 (C), 131.5 (CH), 130.1 (CH), 129.4 (CH), 128.9 (CH), 128.7 (CH), 128.4 (CH), 128.0 (CH), 127.8 (C), 127.1 (CH), 124.3 (CH), 123.3 (C), 113.9 (CH), 103.3 (CH), 98.5 (C), 85.4 (C), 65.5 (OCH_3), 55.5 (CH_2); IR (neat): 3056, 3023, 2926, 2842, 2191, 1718, 1594, 1567, 1508, 1486, 1441, 1332, 1296, 1247, 1167, 1111, 1080, 1025, 934, 830, 756, 687, 614, 570, 527, 474 cm^{-1}; MS (ESI, *m/z*): 408.14 [M+H]$^+$; HRMS (ESI) calcd. for $C_{27}H_{22}NOS$: 408.1417 [M+H]$^+$, found: 408.1411.

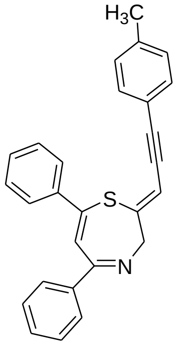

(*Z*)-5,7-Diphenyl-2-[3-(*p*-tolyl)prop-2-yn-1-ylidene]-2,3-dihydro-1,4-thiazepine (TZEP12). A dark brown solid (R_f = 0.56 in 4:1 hexane/ethyl acetate; mp 75.5-76.5 °C). ^{1}H NMR (400 MHz, $CDCl_3$) δ 7.82 (dd, *J* = 7.6, 1.8 Hz, 2H), 7.75 (dd, *J* = 6.5, 3.0 Hz, 2H), 7.47-7.43 (m, 6H), 7.33 (d, *J* = 8.1 Hz, 2H), 7.09 (d, *J* = 8.0 Hz, 2H), 6.94 (s, 1H), 5.79 (s, 1H), 4.88 (s, 2H), 2.33 (s, 3H); ^{13}C NMR (100 MHz, $CDCl_3$), δ 168.0 (C), 150.1 (C), 149.9 (C), 140.0 (C), 138.8 (C), 138.6 (C), 131.4 (CH), 130.7 (CH), 130.2 (CH), 129.2 (CH), 128.9 (CH), 128.6 (CH), 128.0 (CH), 127.8 (CH), 123.9 (C), 120.2 (CH), 104.0 (CH), 98.9 (C), 84.7 (C), 58.5 (CH_2), 21.6 (CH_3); IR (neat): 3023, 2917, 2848, 2345, 2190, 1734, 1563, 1489, 1442, 1294, 1242, 1176, 1097, 1026, 934, 813, 753, 690, 753, 690, 558, 525 cm^{-1}; MS (ESI, *m/z*): 392.14 [M+H]$^+$; HRMS (ESI) calcd. for $C_{27}H_{22}NS$: 392.1468 [M+H]$^+$, found: 392.1481.

Copies of ^{1}H and ^{13}C NMR Spectra

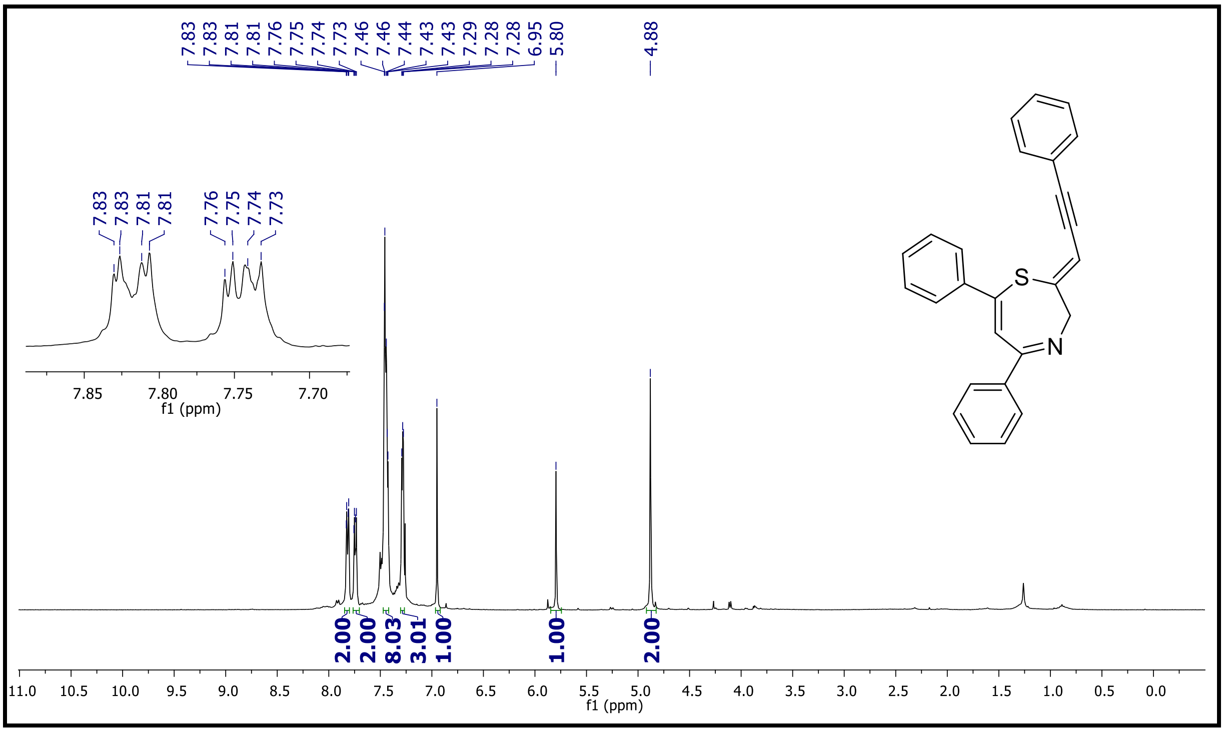

Figure S1. ^{1}H NMR spectrum of compound **TZEP1**.

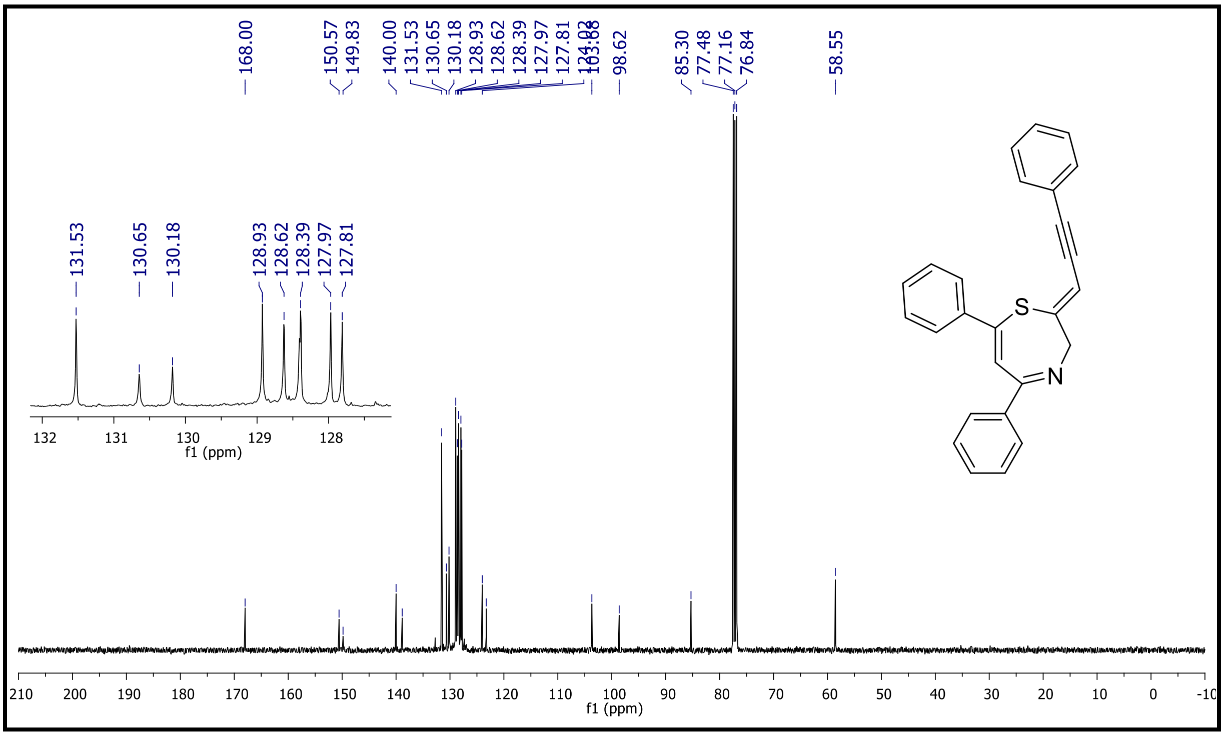

Figure S2. ^{13}C NMR spectrum of compound **TZEP1**.

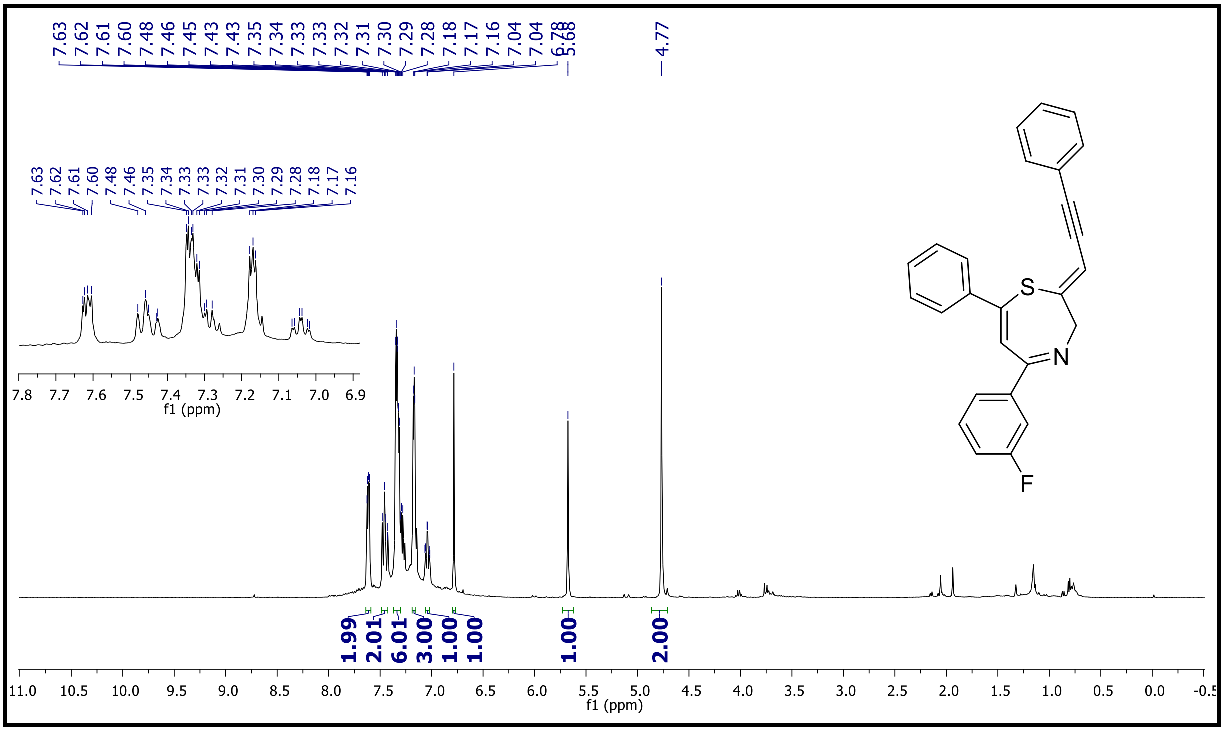

Figure S3. ^{1}H NMR spectrum of compound **TZEP2**.

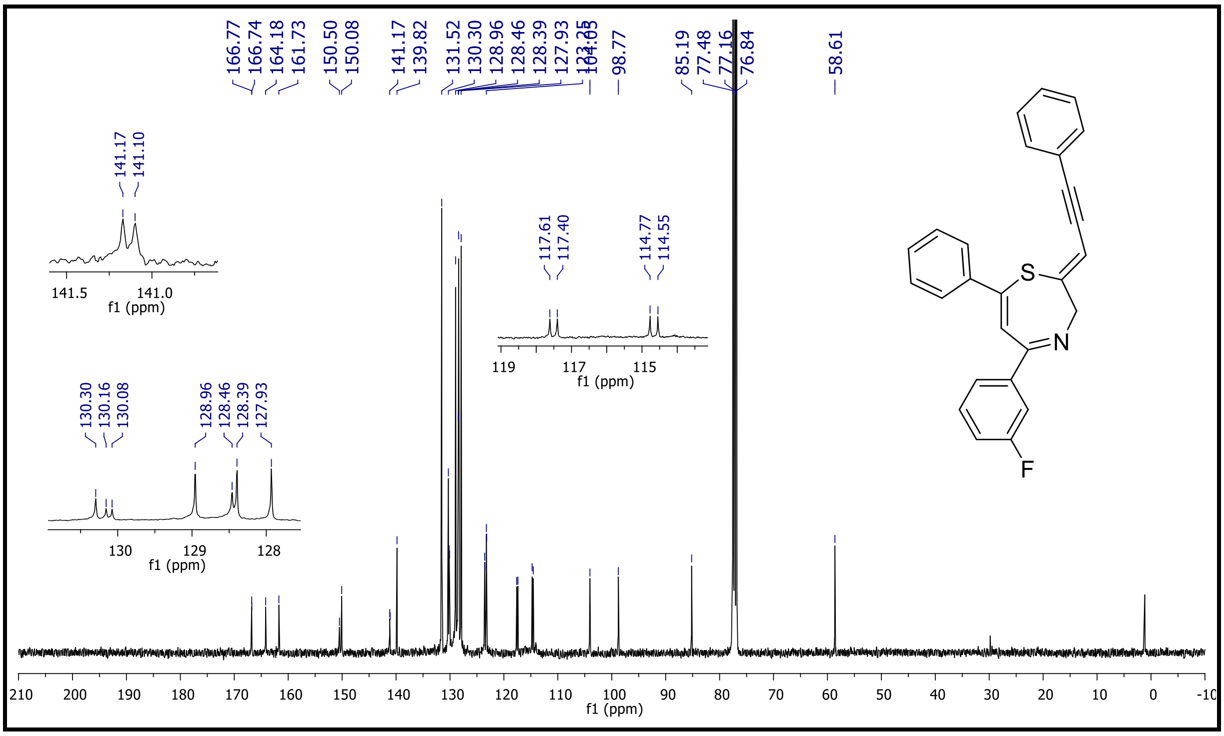

Figure S4. ^{13}C NMR spectrum of compound **TZEP2**.

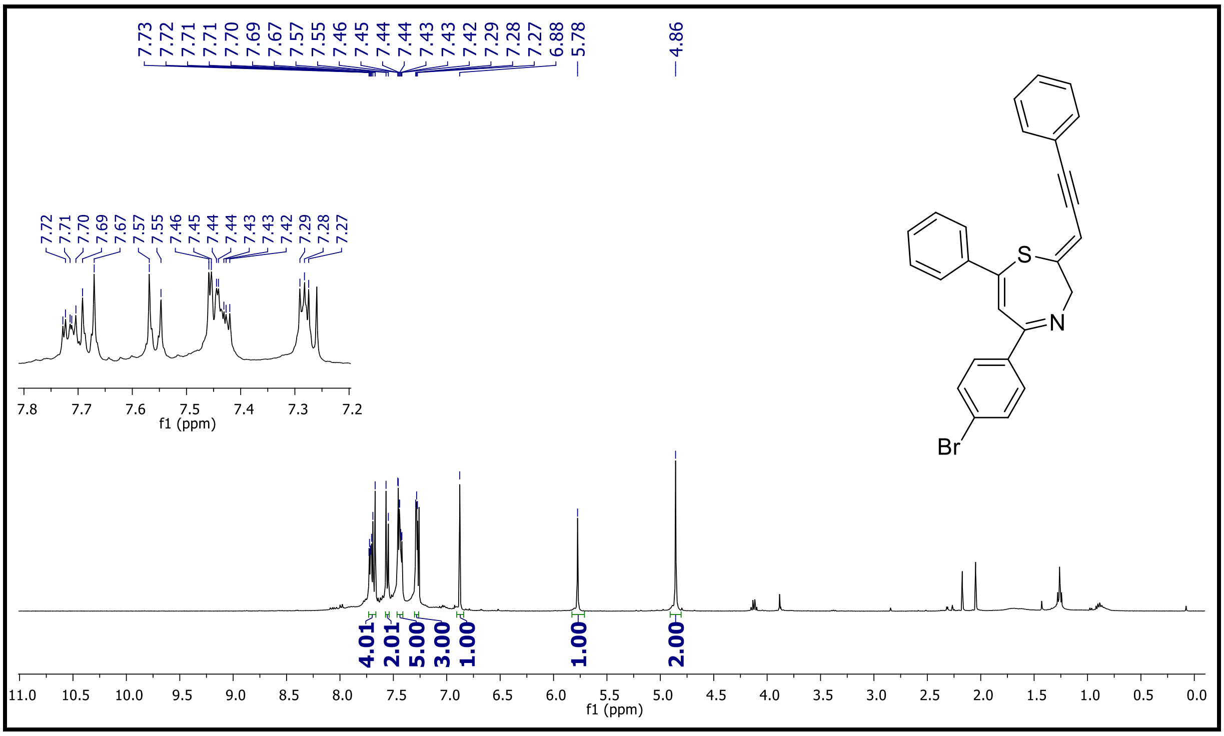

Figure S5. ^{1}H NMR spectrum of compound **TZEP3**.

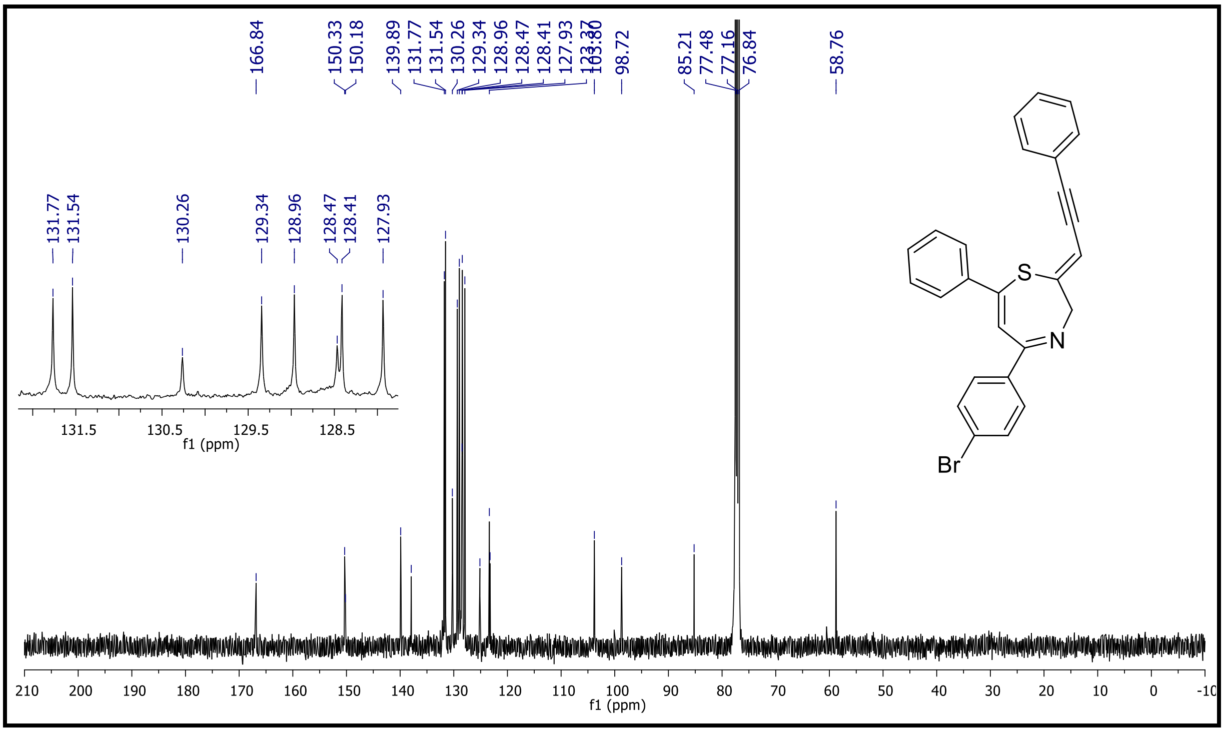

Figure S6. ^{13}C NMR spectrum of compound **TZEP3**.

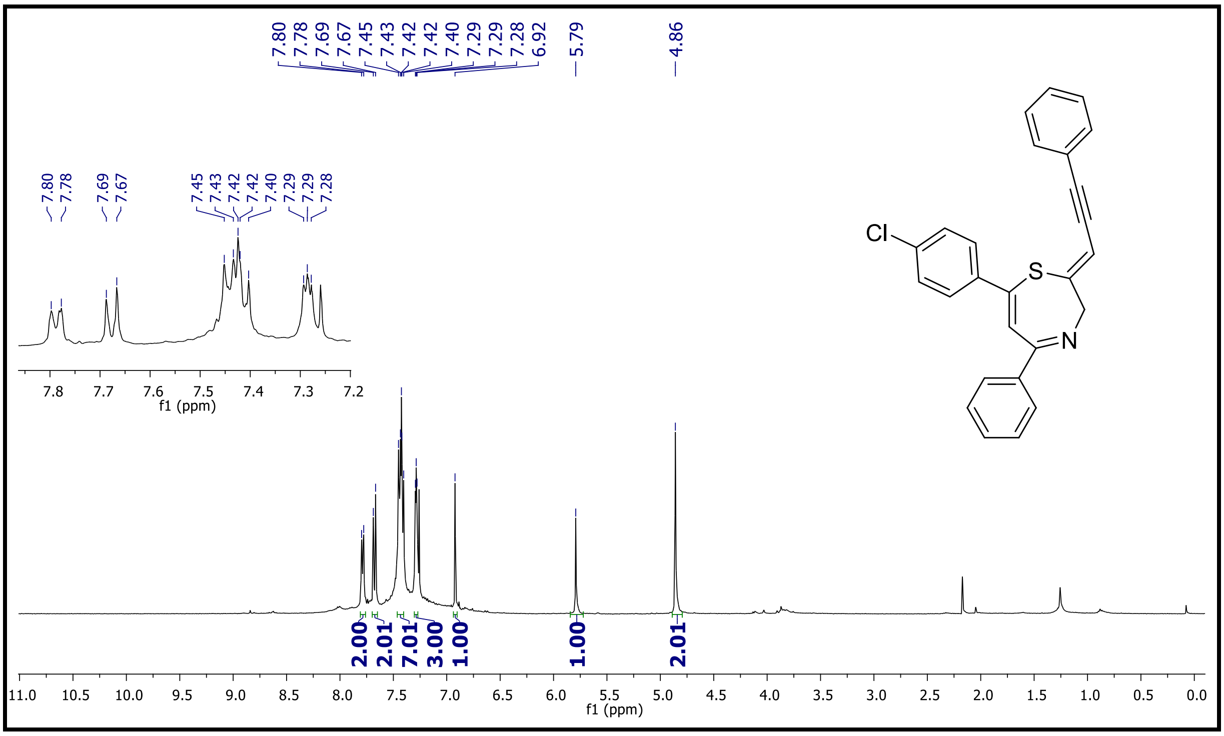

Figure S7. ^{1}H NMR spectrum of compound **TZEP4**.

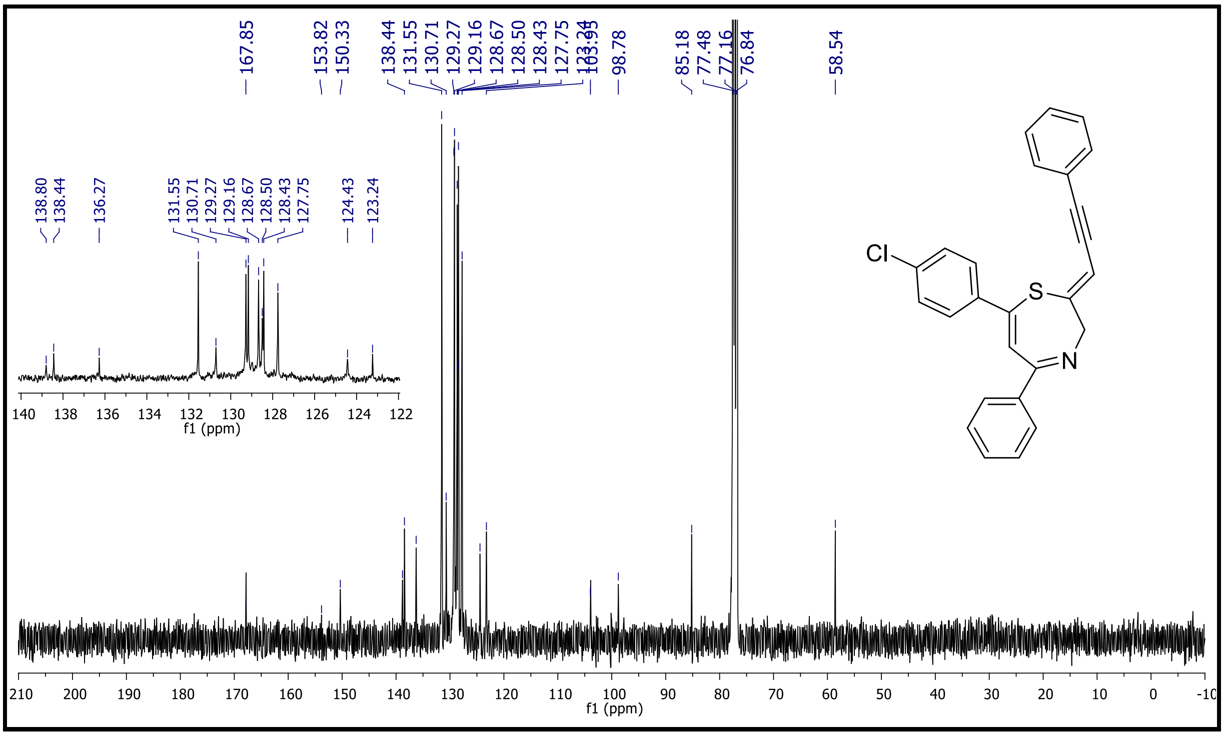

Figure S8. ^{13}C NMR spectrum of compound **TZEP4**.

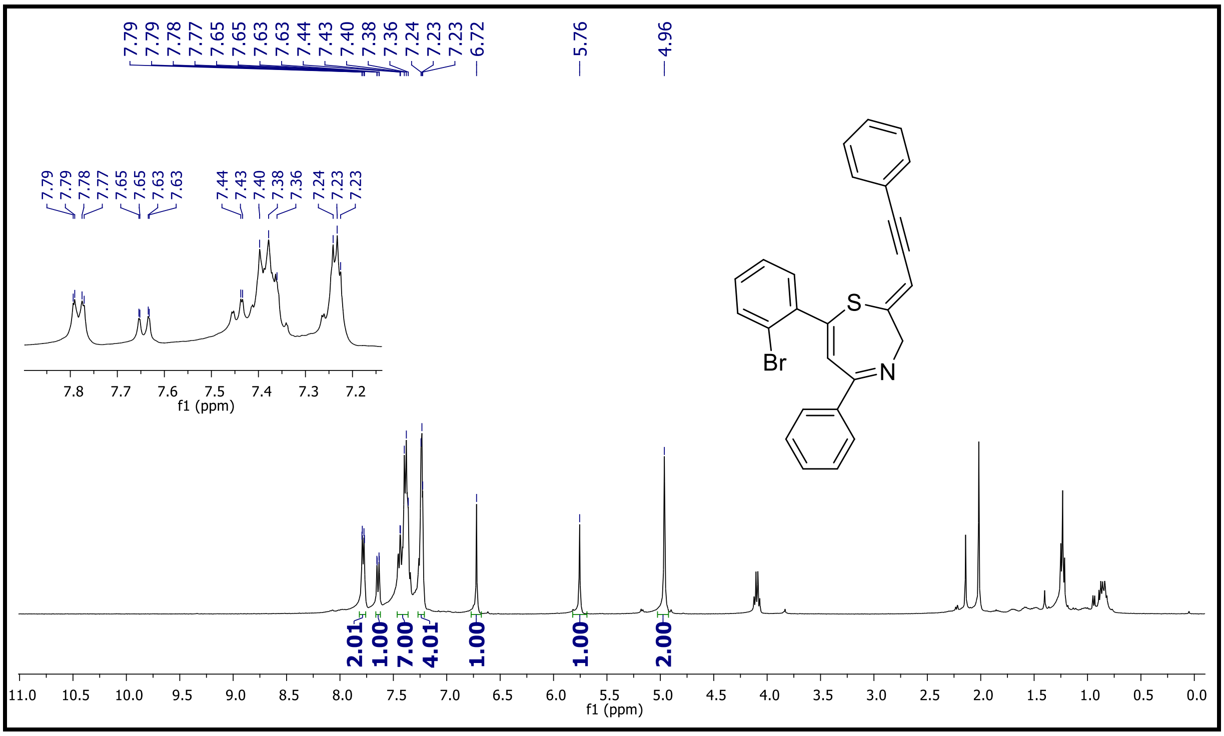

Figure S9. ^{1}H NMR spectrum of compound **TZEP5**.

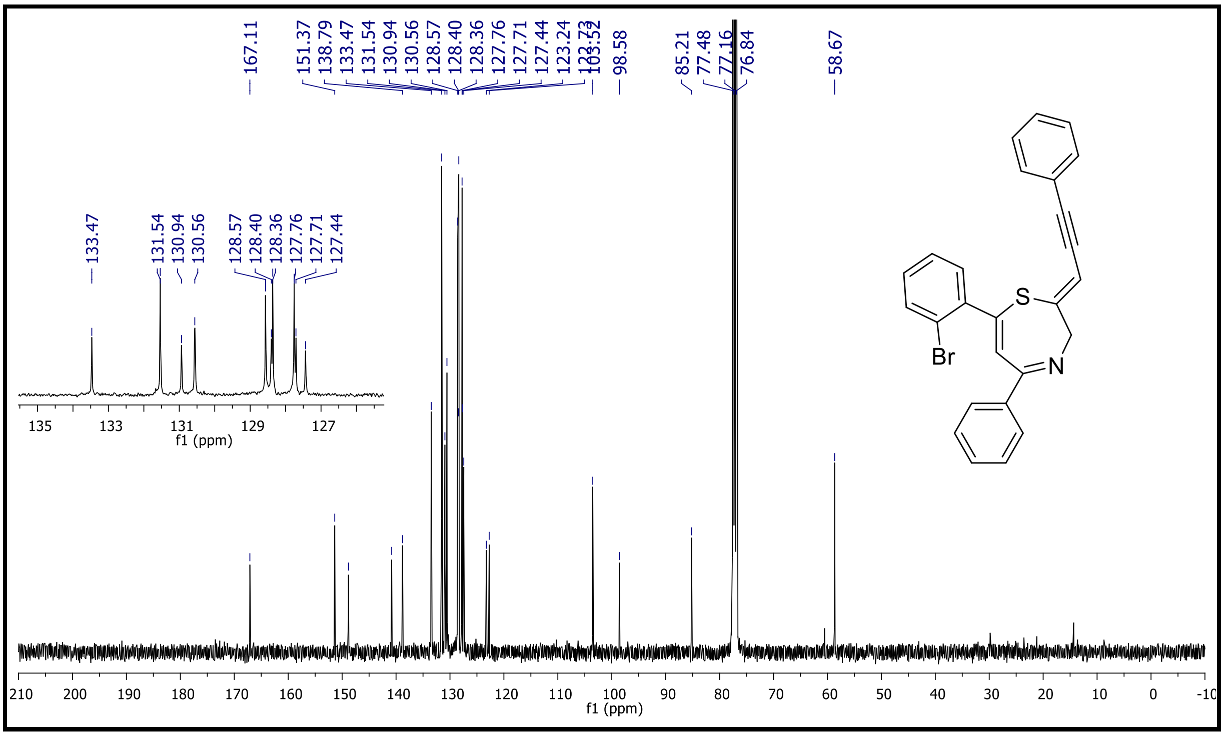

Figure S10. ^{13}C NMR spectrum of compound **TZEP5**.

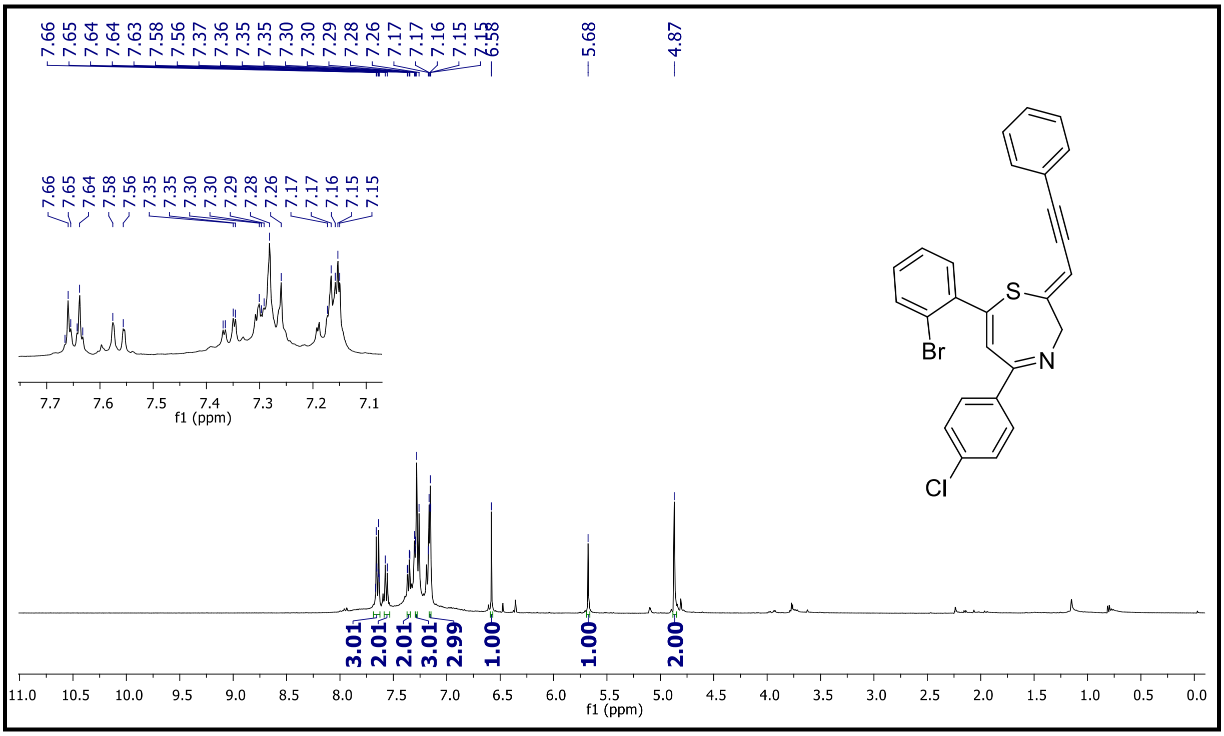

Figure S11. ^{1}H NMR spectrum of compound **TZEP6**.

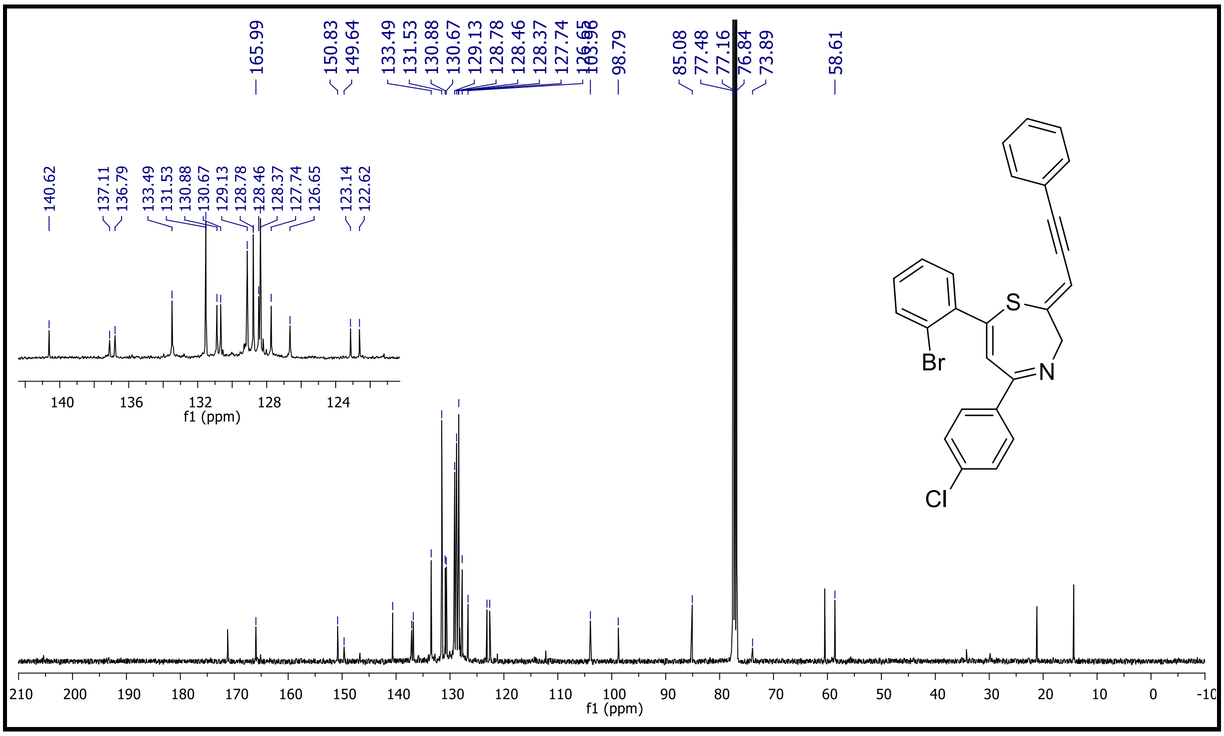

Figure S12. ^{13}C NMR spectrum of compound **TZEP6**.

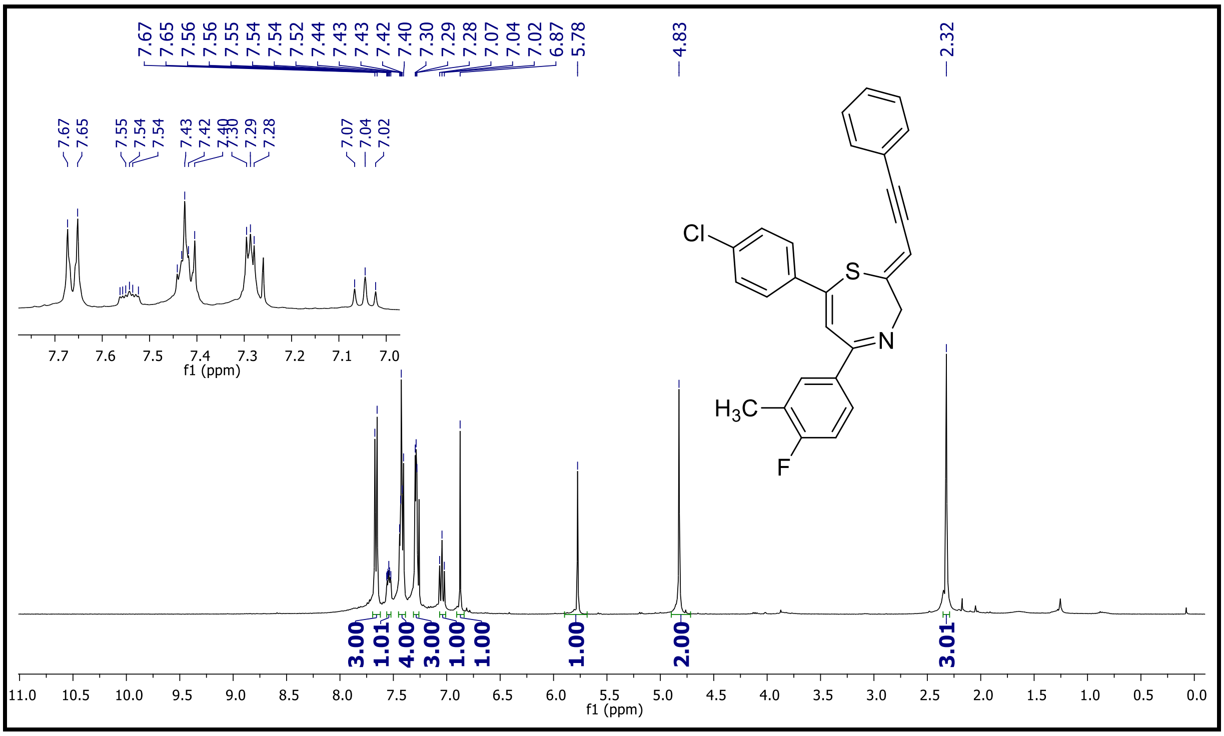

Figure S13. ^{1}H NMR spectrum of compound **TZEP7**.

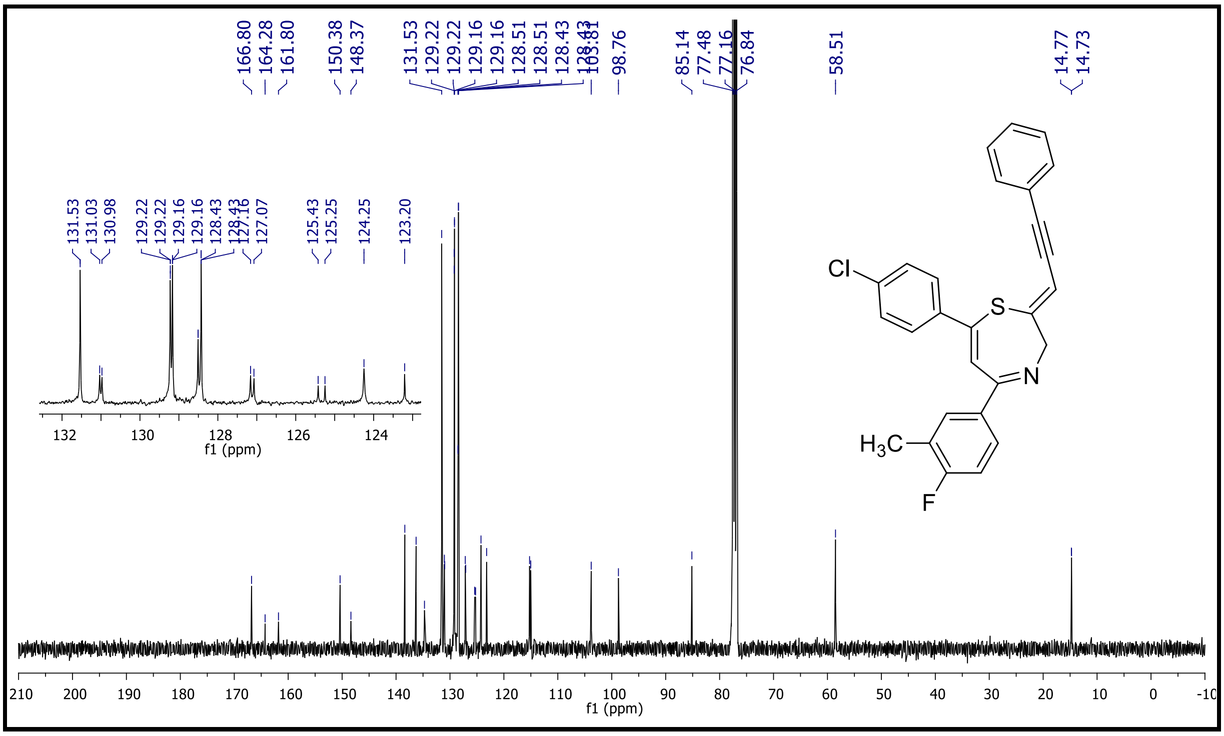

Figure S14. ^{13}C NMR spectrum of compound **TZEP7**.

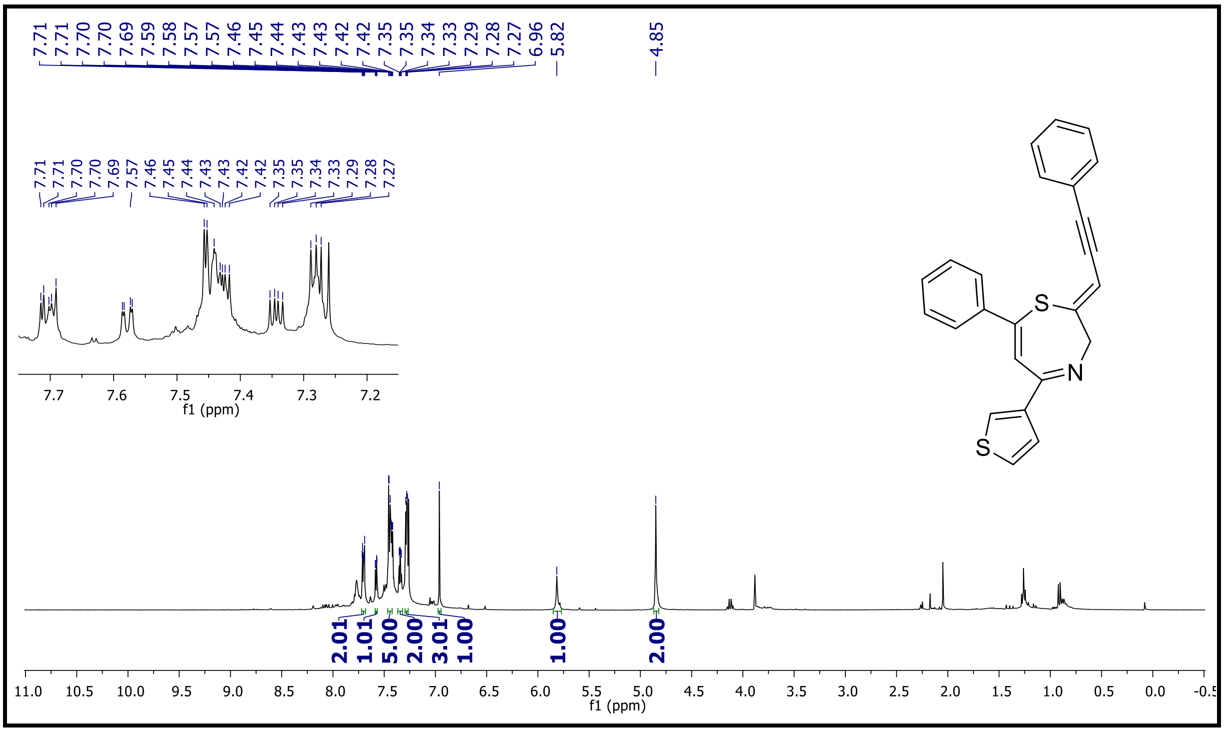

Figure S15. ^{1}H NMR spectrum of compound **TZEP8**.

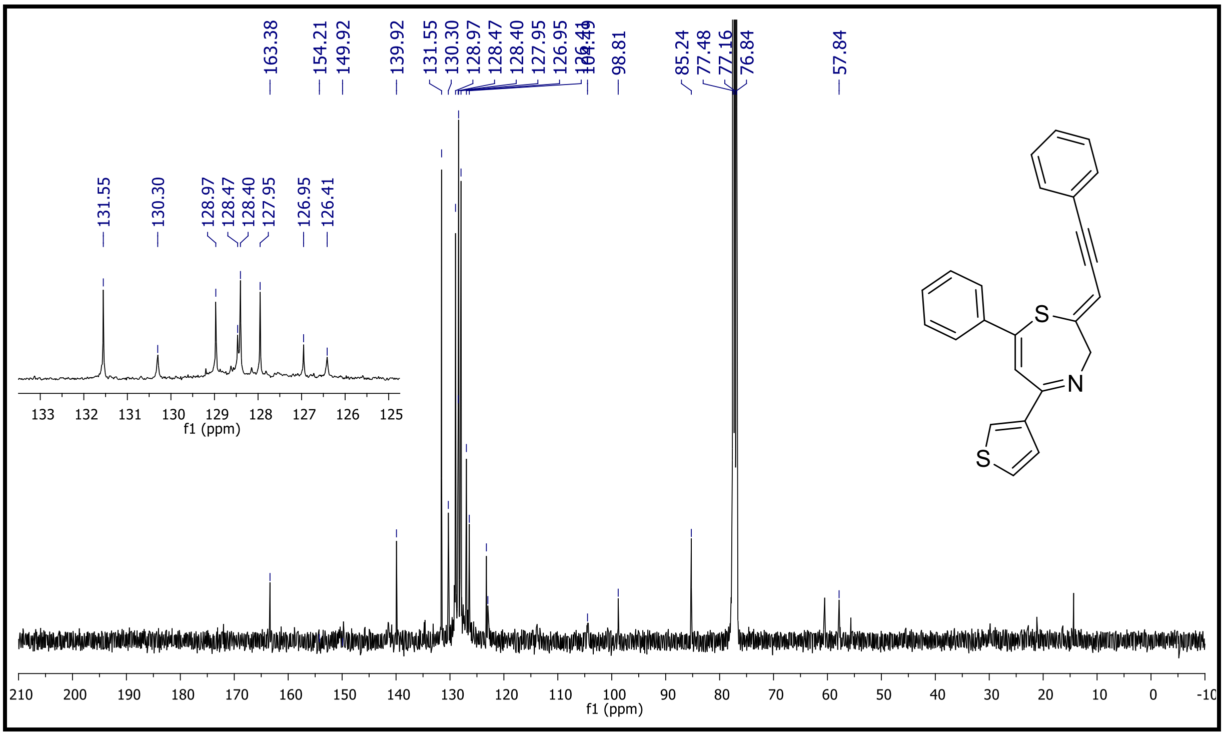

Figure S16. ^{13}C NMR spectrum of compound **TZEP8**.

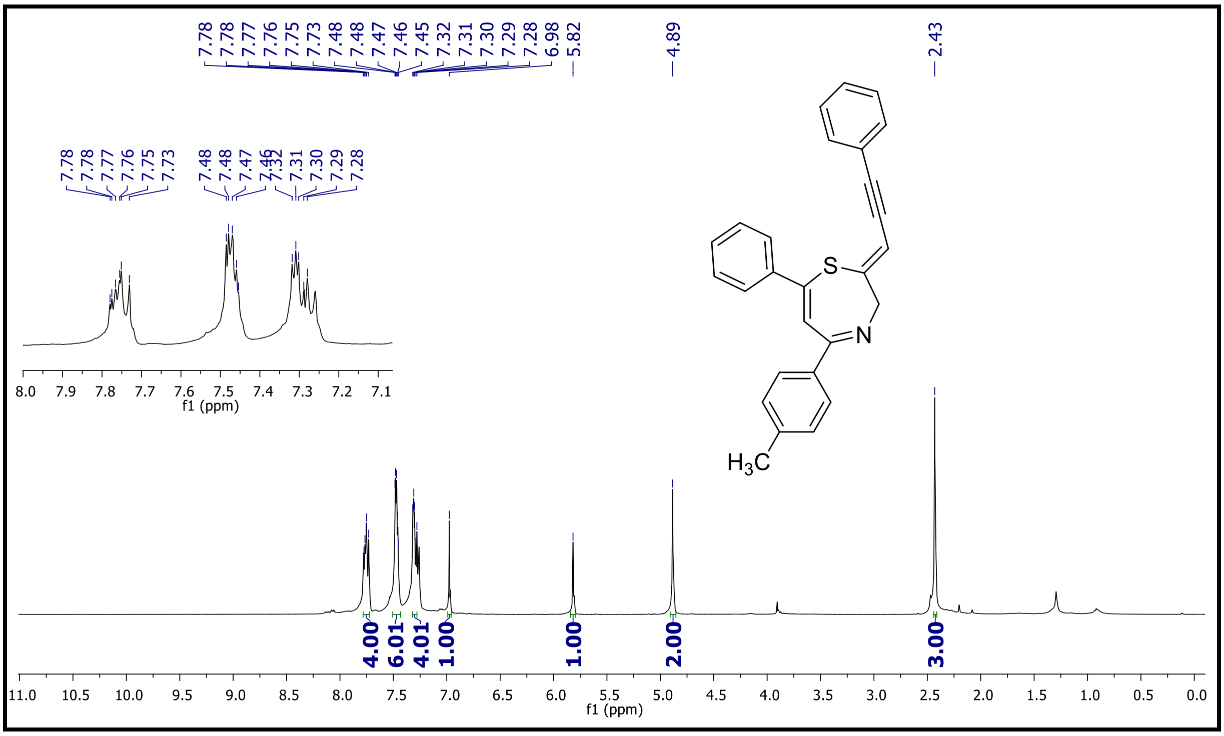

Figure S17. ^{1}H NMR spectrum of compound **TZEP9**.

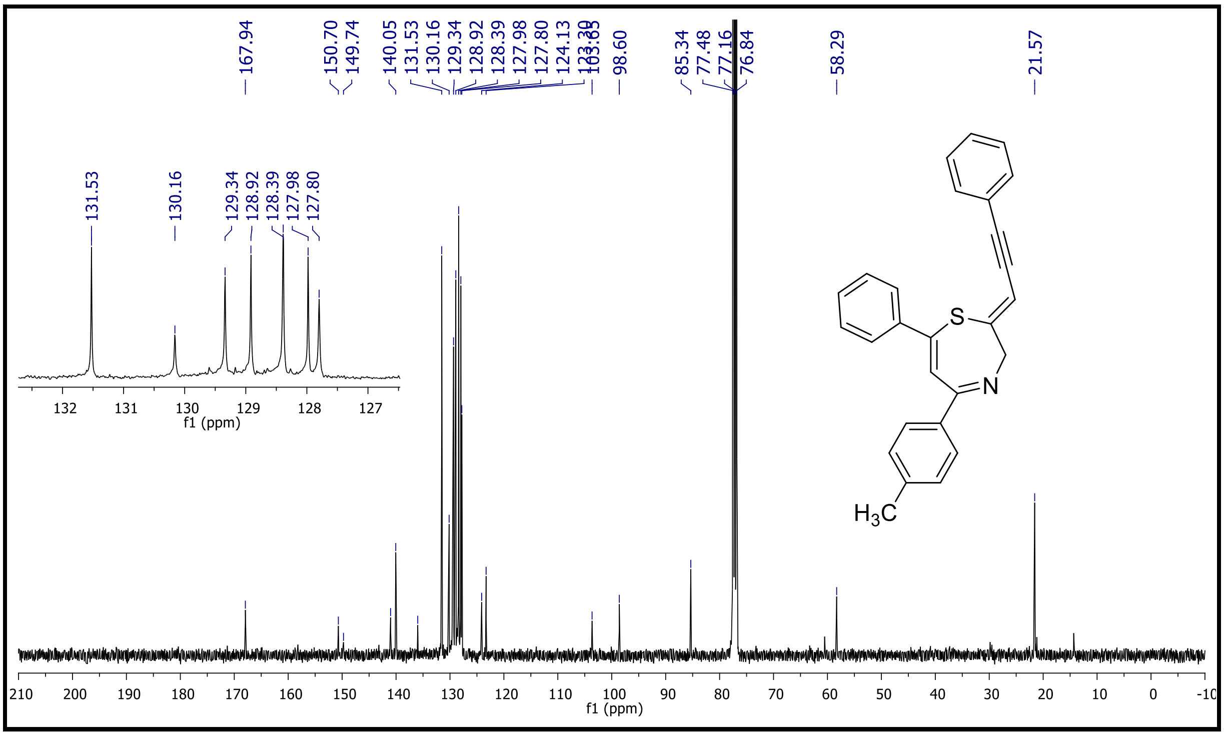

Figure S18. ^{13}C NMR spectrum of compound **TZEP9**.

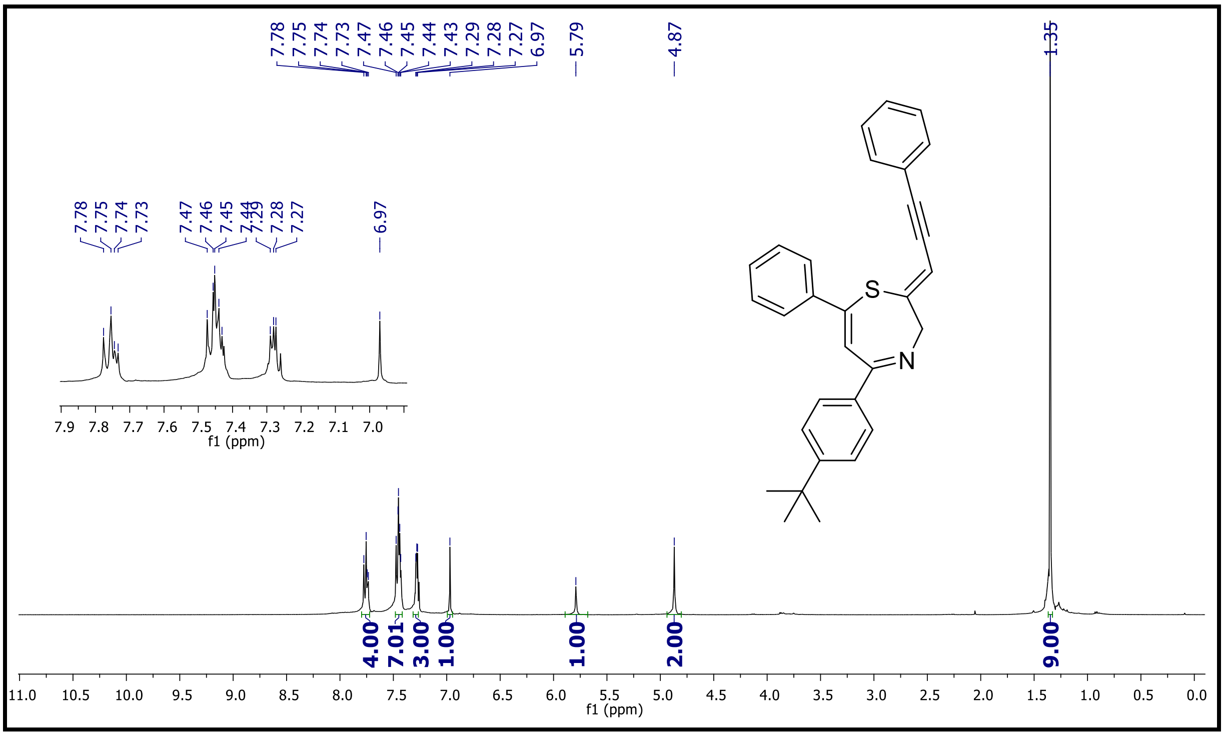

Figure S19. ^{1}H NMR spectrum of compound **TZEP10**.

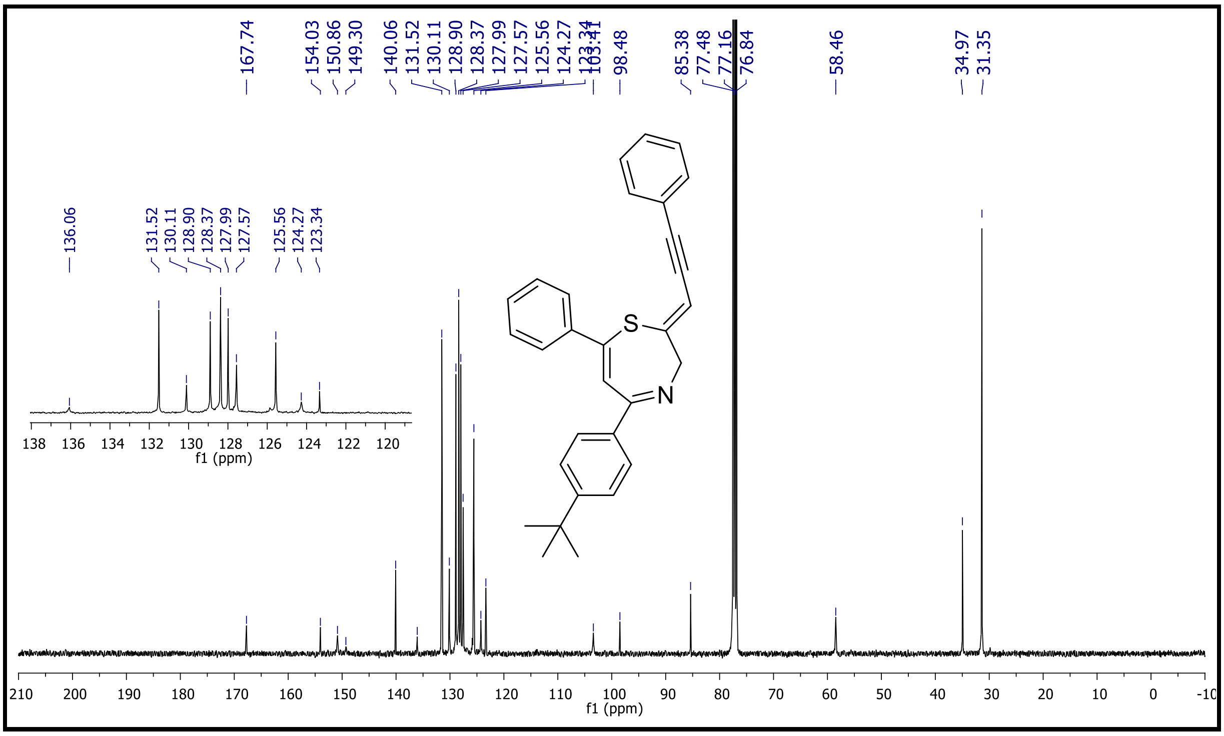

Figure S20. ^{13}C NMR spectrum of compound **TZEP10**.

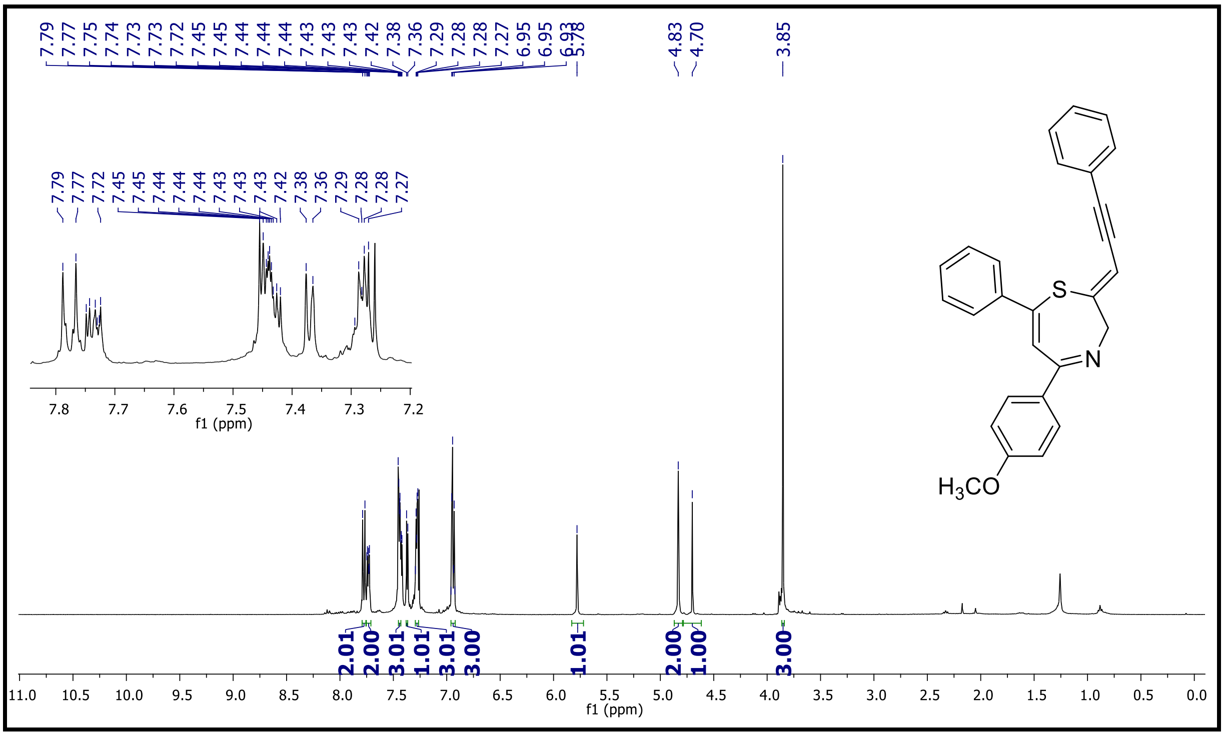

Figure S21. ^{1}H NMR spectrum of compound **TZEP11**.

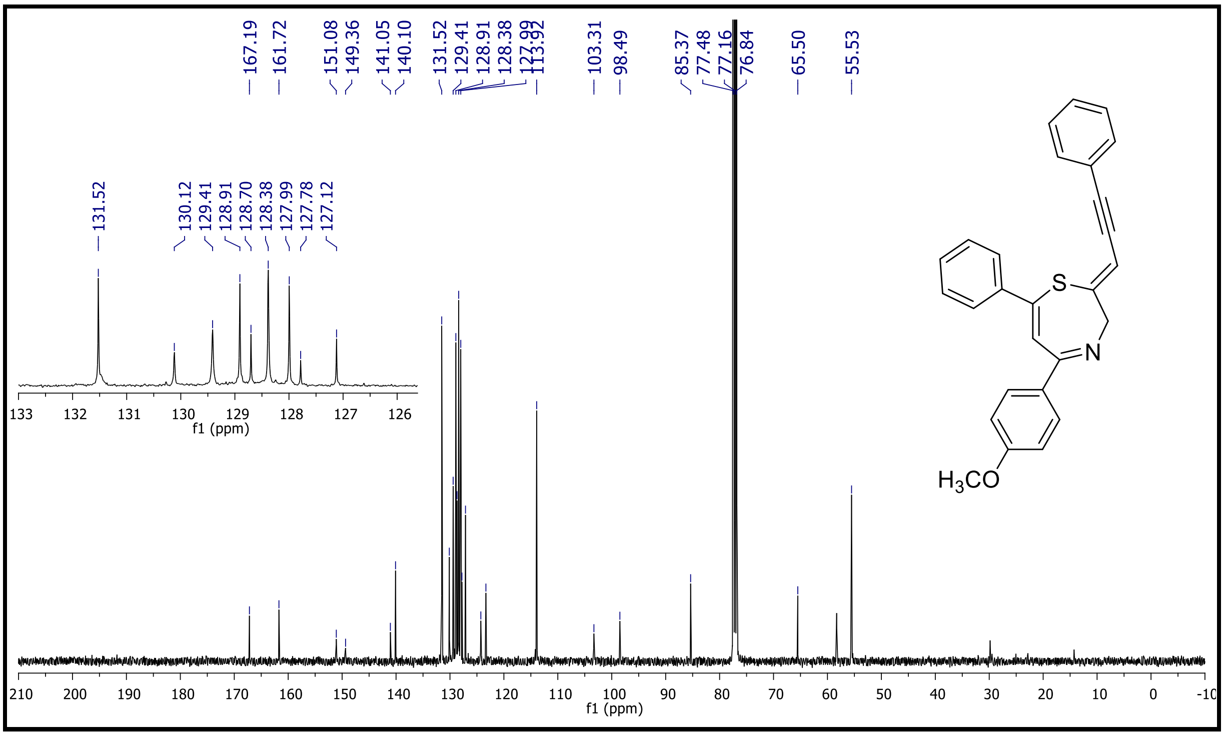

Figure S22. ^{13}C NMR spectrum of compound **TZEP11**.

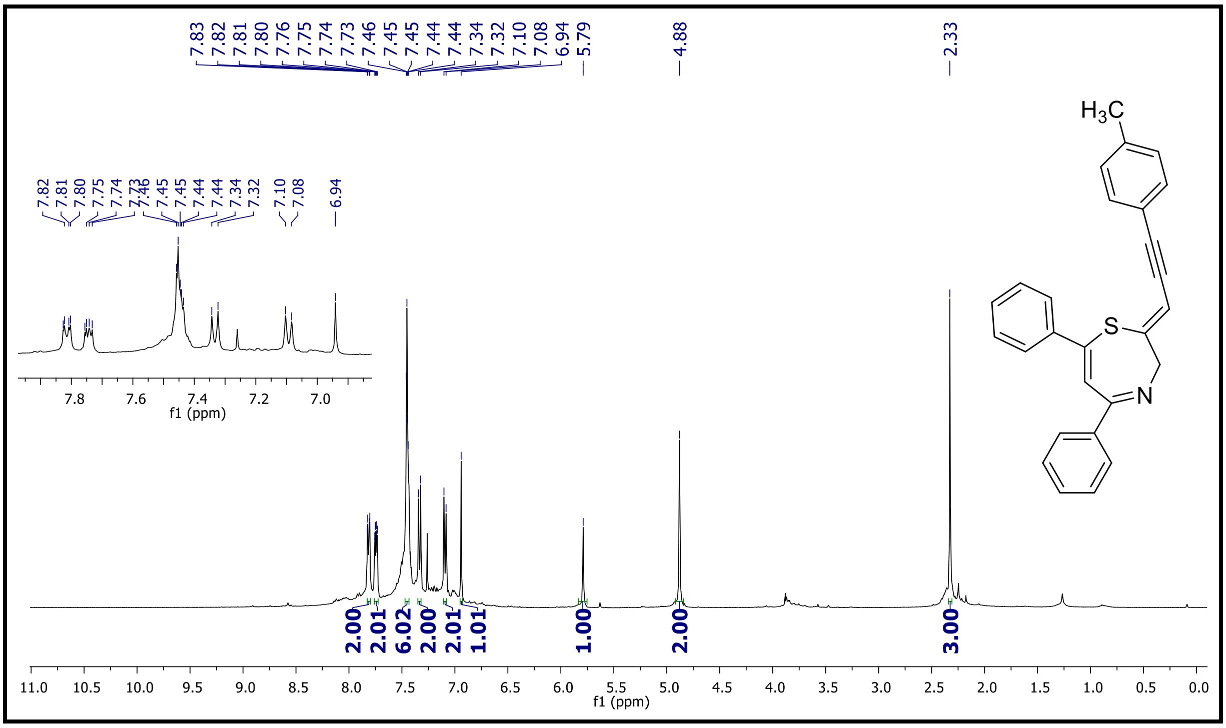

Figure S23. ^{1}H NMR spectrum of compound **TZEP12**.

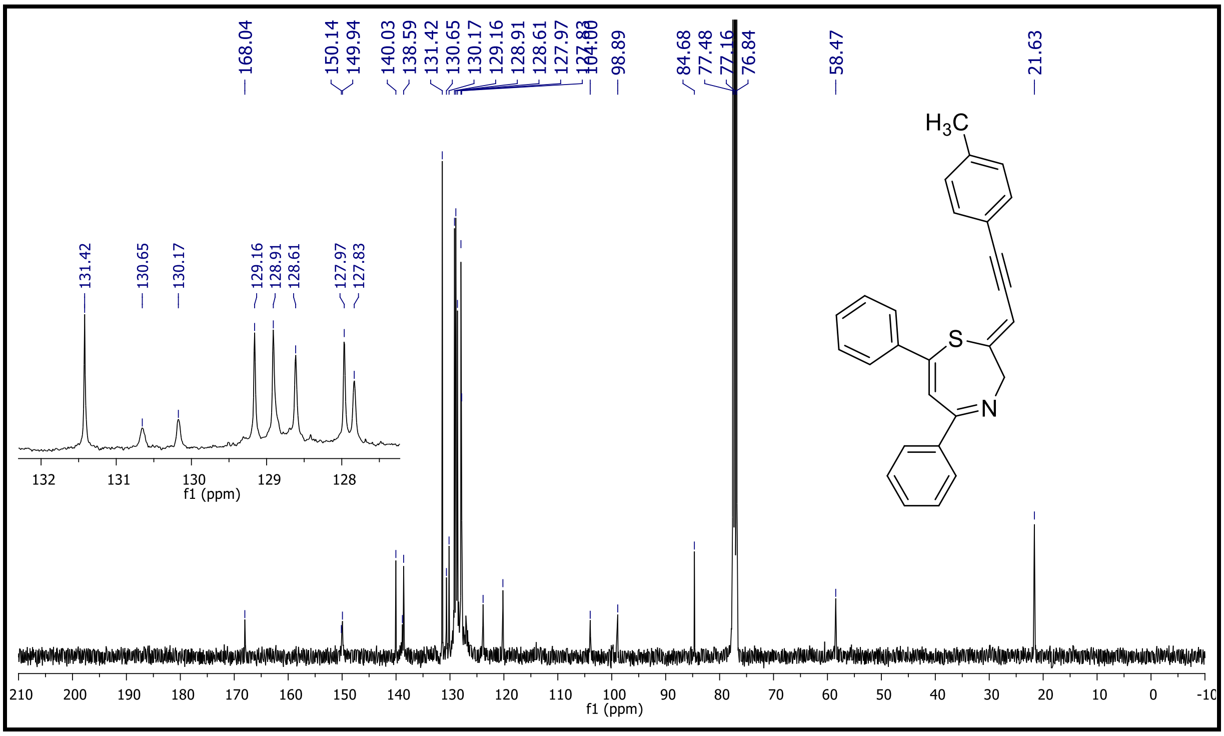

Figure S24. ^{13}C NMR spectrum of compound **TZEP12**.

References

1. Yilmaz, E. S.; Zora, M. A New Strategy for the Synthesis of 4-Propargyl-Substituted 1*H*-Pyrroles from *N*-(5-phenyl-2,4-pentadiynyl) β-Enaminones. *ChemistrySelect* **2019**, *4*, 11043–11047. https://doi.org/10.1002/slct.201902759

2. Yilmaz, E. S.; Zora, M. A Facile One-Pot Synthesis of 2-(Prop-2-yn-1-ylidene)-2,3-dihydro-1,4-thiazepines. *Synth. Commun.* **2021**, *51*, 709–719. https://doi.org/10.1080/00397911.2020.1850795
